# Supplementary material for: Coral Lipids
Source: Mar Drugs. 2023 Oct 15;21(10):539. doi: 10.3390/md21100539 (PMC10608786; doi:10.3390/md21100539)
Supplement: Supplementary file 1 [file marinedrugs-21-00539-s001.zip › marinedrugs-2597168-supplementary.pdf]

Supporting material.

Coral Lipids

Table S1. The total lipid content in corals collected at the Great Barrier Reef (Australia) at the depth of 4 m in July [33].

No.	Taxonomic group	Lipid content (mg/g of colony)
	**Schleractinia**	
	Acroporidae	
1*	*Acropora hyacinthus*	3.8
2	*Acropora echinata*	9.7
3	*Astreopora ocellata*	13.1
	Agaraciidae	
4	*Gardineroseris planulata*	6.3
5	*Pachyseris rugosa*	1.8
	Capyophylliidae	
6	*Physosyra lichtensteini*	23.2
	Dendrophyllidae	
7	*Tubastraea coccinea*	26.3
8	*Dendrophyllia* cf. *micranthus*	29.3
9	*Turbinaria* cf. *frondens*	5.5
10	*Turbinaria* cf. *sinensis*	7.1
	Faviidae	
11	*Caulastrea furcata*	15.7
12	*Hydnophora rigida*	22.6
13	*Hydnophora exasa*	8.6
14	*Echinopora horrid*	12.3
15	*Echinopora* cf. *hirsutissima*	4.6
16	*Favia favus*	2.2
17	*Leptoria phyrgia*	3.5
18	*Platygyra sinensis*	1.9
19	*Hydrophora* sp.	5.0
20	*Favia stelligera*	1.3
	Fugiidae	
21	*Fungia actiniformis*	14.0
22	*Fungia fungites*	16.2
23	*Herpolitha limax*	11.6
	Merulinidae	
24	*Clavarina scrabicula*	2.7
	Mussidae	
25	*Lobophyllia corymbosa*	4.2

	Oculinidae	
26	*Galaxea fascicularis*	8.9
27	*Galaxea* sp.	9.0
	Pectinidae	
28	*Echinophyllia aspera*	2.5
29	*Echinophyllia* sp.	4.5
30	*Pectinia lactuca*	8.8
31	*Oxypora lacera*	10.5
	Pocilloporidae	
32	*Stylophora pistillata*	4.0
33	*Pocillipora damicornus*	5.8
34	*Seriatopora hystrix*	13.8
	Porirdae	
35	*Porites andrewsi*	13.1
36	*Goniopora gracilis*	45.2
	Thamnasteriidae	
37	*Psammocora contiqua*	6.6
	**Tubipora**	
	Octacollaria	
38	*Tubipora musica*	83.0

* Coral numbers to be used in other tables.

Table S2. Lipid composition (% of total) in the hard and soft corals from coastal waters of Vietnam (the South China Sea) [68].

No	Species	PL	ST	FFA	TG	MADAG	WE+SE	Others
1	*Acropora cytherea*	16.1	5.7	1.6	31.8	3.6	38.9	2.3
2	*Acropora cytherea*	24.1	7.6	1.1	19.1	1.9	42.8	3.4
3	*Acropora acuminate*	14.4	3.8	2.5	30.4	2.6	44.3	2.0
4	*Acropora microphthalma*	18.5	5.5	2.9	28.0	0.9	42.2	2.0
5	*Acropora hacinthum*	22.6	7.9	1.5	23.7	12.5	30.1	1.7
6	*Acropora nobilis*	16.9	4.4	1.2	21.5	2.8	45.6	7.6
7	*Acropora grandis*	11.9	4.6	1.3	31.4	8.8	40.4	1.6
8	*Acropora samoensis*	23.7	10.0	1.2	28.6	1.0	33.9	1.6
9	*Acropora humilis*	12.9	4.8	0.9	33.2	3.2	43.9	1.1
10	*Astreopora ocelata*	12.5	5.0	0.9	32.9	6.2	41.1	1.4
11	*Pavona frondifera*	25.3	5.8	1.1	26.3	4.8	35.7	1.0
12	*Pavona frondifera*	17.4	5.2	1.1	31.5	6.9	36.4	1.5
13*	*Balanophyllia sp.*	25.4	12.7	1.9	10.4	5.4	42.2	2.0
14*	*Tubastrea aurea*	32.2	10.6	1.7	9.0	3.1	40.3	3.1
15	*Turbinaria mesenterina*	15.3	7.4	2.0	15.2	4.3	53.6	2.2
16	*Turbinaria mesenterina*	11.6	6.8	1.1	22.2	5.9	51.3	1.1
17	*Turbinaria peltata*	12.5	5.9	1.3	29.1	6.5	43.0	1.7
18	*Turbinaria peltata*	10.2	3.9	1.2	30.8	3.0	49.4	1.5

19	*Turbinaria peltata*	12.1	3.9	0.6	29.8	8.1	44.6	0.9
20	*Euphyllia ancora*	11.1	3.7	0.5	43.1	8.4	32.4	0.8
21	*Euphyllia ancora*	19.5	5.4	0.9	38.4	6.2	29.6	0.0
22	*Cyphastrea chalcidicum*	24.2	5.0	1.5	8.8	1.2	57.5	1.8
23	*Cyphastrea serailia*	4.0	3.9	1.2	27.2	7.1	55.6	1.0
24	*Goniastrea chinensis*	10.4	2.2	0.8	27.4	3.1	55.8	0.3
25	*Goniastrea pectinata*	16.0	5.5	0.5	25.7	4.2	46.8	1.3
26	*Favia lizardensis*	7.6	3.2	1.5	25.3	4.2	56.4	1.8
27	*Favia maxima*	8.8	5.7	1.2	26.1	2.6	54.4	1.2
28	*Favia maxima*	11.8	5.2	0.7	13.8	1.4	66.4	0.7
29	*Favia maritima*	17.5	6.1	0.6	16.6	3.4	54.9	0.9
30	*Favia favus*	10.1	10.6	1.5	32.4	14.5	29.0	1.9
31	*Favites abdita*	17.6	6.5	0.6	13.8	1.6	58.7	1.2
32	*Favites chinensis*	4.9	4.4	3.0	20.0	1.7	62.8	3.2
33	*Favites flexuosa*	12.1	5.9	0.8	20.8	2.7	56.6	1.1
34	*Fungia scutaria*	27.2	5.3	0.8	7.8	0.4	58.1	0.4
35	*Fungia fungites*	15.8	4.0	0.4	17.8	2.8	58.8	0.4
36	*Fungia scutaria*	15.4	4.2	0.5	25.8	5.1	48.5	0.5
37	*Fungia scrupusa*	14.8	5.7	0.6	19.1	3.8	55.5	0.5
38	*Cycloseris costulata*	12.8	3.3	0.3	16.9	4.1	62.4	0.2
39	*Litophyllon undulatum*	20.3	5.4	0.9	20.1	4.7	47.5	1.1
40	*Podobacia crustacean*	18.8	4.5	0.5	16.7	2.4	57.1	0.0
41	*Sandalolitha robusta*	18.4	5.1	0.7	15.1	2.1	58.5	0.1
42	*Echinophyllia orphensis*	7.4	4.4	1.4	21.1	2.4	61.9	1.4
43	*Echinophyllia ehinata*	19.8	5.3	0.8	8.2	2.7	62.4	0.8
44	*Goniopora lobata*	18.4	6.0	0.8	10.0	6.7	57.6	0.5
45	*Goniopora stokesi*	10.5	4.3	0.9	20.7	5.5	57.2	0.9
46	*Porites lutea*	14.9	5.6	0.6	39.1	9.3	29.3	1.2
47	*Porites lutea*	19.3	7.9	1.6	18.1	6.3	44.6	2.2
48	*Porites solida*	15.7	7.5	0.8	39.7	9.1	26.4	0.8
49	*Galaxea fascicularis*	23.9	7.6	0.5	25.7	3.1	35.9	3.3
50	*Millepora dichotoma*	19.2	5.6	0.7	22.8	17.4	26.7	7.6
51	*Millepora platyphylla*	15.1	5.4	0.9	22.0	18.6	37.1	0.9
52	*Heliopora coerulea*	18.3	5.7	0.8	20.1	20.1	33.9	1.1
53**	*Carijoa riisei*	32.8	6.1	1.6	12.8	12.1	32.6	2.0
54**	*Carijoa riisei*	37.7	7.8	1.8	17.8	19.6	13.1	2.2
55**	*Klyxum molle*	27.7	5.5	1.6	4.9	14.1	44.4	1.8
56	*Cladiella laciniosa*	19.9	5.4	1.5	3.5	12.5	55.5	1.7
57	*Cladiella subtilis*	31.4	9.0	1.6	8.6	20.7	26.9	1.8
58	*Cladiella pachyclados*	43.7	9.8	2.3	3.7	15.6	22.5	2.4
59	*Lobophytum michaelae*	20.3	14.7	1.5	6.6	25.3	30.1	1.5

60	*Lobophytum ransoni*	33.6	8.1	1.5	10.0	20.8	24.4	1.6
61	*Lobophytum batarum*	24.3	5.6	0.8	7.4	16.0	38.0	7.9
62	*Lobophytum crassum*	35.1	11.9	1.9	2.4	14.4	32.7	1.6
63	*Lobophytum* sp.	13.7	12.8	1.8	10.2	20.5	39.9	1.1
64	*Sarcophyton crassum*	22.4	5.9	0.7	9.6	24.8	35.8	0.8
65	*Sarcophyton ehrenbergi*	36.5	8.3	2.1	3.5	13.3	34.1	2.2
66	*Sarcophyton* cf. *glaucum*	16.0	3.9	0.5	9.5	21.5	48.1	0.5
67	*Sarcophyton regulare*	34.9	9.0	1.5	11.3	14.6	27.3	1.4
68	*Sarcophyton cinereum*	40.0	9.1	1.5	5.0	15.6	26.9	1.9
69	*Sarcophyton spongiosum*	22.4	7.0	1.4	9.7	12.6	35.4	11.5
70	*Sarcophyton* sp.	18.6	4.1	0.8	7.8	19.5	48.1	1.1
71	*Sinularia* cf. *robusta*	33.2	4.9	2.0	4.6	18.8	33.6	2.9
72	*Sinularia* aff. *exilis*	24.4	4.9	1.5	6.0	25.2	36.0	2.0
73	*Sinularia brassica*	32.6	6.9	1.4	4.8	17.1	35.7	1.5
74	*Sinularia brassica*	34.6	6.9	1.7	5.3	14.2	35.7	1.6
75	*Sinularia erecta*	30.0	10.6	1.5	9.7	19.9	25.9	2.4
76	*Sinularia* aff. *polydactila*	37.4	9.6	1.5	9.1	16.7	23.3	2.4
77	*Sinularia siaesensis*	18.9	6.4	1.2	5.3	30.7	35.8	1.7
78	*Sinularia siaesensis*	21.4	2.7	0.4	3.7	18.0	51.9	1.9
79	*Sinularia. gibberosa*	25.5	5.0	1.3	7.9	32.7	25.5	2.1
80	*Sinularia* sp.	30.0	6.7	2.1	11.1	19.3	28.7	2.1
81	*Sinularia. polydactila*	45.9	18.0	1.4	8.6	4.1	20.6	1.4
82	*Sinularia flexibilis*	50.7	20.3	1.1	4.2	3.4	19.2	1.1
83*	*Dendronephthya* cf. *cervicornis*	45.9	17.6	1.3	8.9	6.4	18.5	1.4
84*	*Dendronephthya* sp.	31.7	5.8	1.7	9.7	17.5	31.7	1.9
85*	*Dendronephthya* cf. *pulchella*	29.2	6.3	1.2	10.4	14.6	36.8	1.5
86	*Lemnalia* cf. *exilis*	19.7	6.1	1.4	21.4	11.0	38.9	1.5
87	*Lemnalia* cf. *peristyla*	28.7	8.8	1.7	14.6	14.2	30.1	1.9
88	*Lemnalia capnelliformis*	43.3	13.4	1.6	21.2	13.0	7.0	0.5
89	*Nephthea* sp.	43.0	7.1	2.3	8.6	7.5	27.2	4.3
90	*Nephthea* sp.	40.3	20.8	1.4	3.4	6.7	25.0	2.4
91*	*Siphonogorgia variabilis*	42.9	17.0	2.0	4.4	7.5	26.0	0.2
92*	*Siphonogorgia* cf. *harrisoni*	30.1	11.2	1.5	21.4	16.8	17.5	1.5
93*	*Siphonogorgia* cf. *harrisoni*	35.6	11.1	1.0	18.7	17.0	15.5	1.1
94*	*Annella mollis*	41.9	14.7	2.1	5.5	5.4	23.5	6.9
95*	*Annella mollis*	44.0	13.9	2.5	14.1	7.0	15.8	2.7
96*	*Mopsella* sp.	30.0	9.2	1.7	13.9	20.0	23.5	1.7
97*	*Mopsella* cf. *spinosa*	40.2	12.3	1.9	6.0	11.1	26.0	2.5
98*	*P.* cf. *minor*	36.4	7.3	1.5	6.5	9.5	33.7	5.1
99*	*Menella* cf. *praelonga*	42.2	14.3	1.4	5.7	10.7	24.3	1.4
100*	*Menella* cf. *praelonga*	47.5	14.2	3.0	8.5	9.9	13.7	3.2

101*	*Menella flora*	27.1	9.7	1.5	7.1	8.0	46.6	0.0
102*	*Echinogorgia* cf. *gracillima*	31.6	9.7	1.1	9.7	34.6	12.1	1.2
103*	*Paracis* cf. *horrida*	18.6	5.9	0.6	13.4	51.9	9.3	0.3
104	*Hicksonella princeps*	19.3	5.1	1.0	11.0	14.1	48.5	1.0
105	*Hicksonella princeps*	27.1	10.3	1.5	12.3	13.9	33.3	1.6
106*	*Viminella* cf. *petila*	53.7	11.9	1.6	7.4	5.4	19.0	1.0
107*	*Viminella* cf. *crassa*	15.8	5.9	2.0	5.8	18.0	50.3	2.2
108**	*Narella* sp.	41.1	14.0	1.1	13.2	9.8	18.8	2.0
109**	Plexauridae spp. 1	58.8	8.6	1.4	11.2	5.7	12.9	1.4
110**	Plexauridae spp. 2	25.6	10.6	2.4	1.9	7.5	49.6	2.4

* Species without zooxanthellae. ** No data on zooxanthellae presence. Coral numbers to be used in other tables.

Table S3. Hydrocarbon's composition (weight % of the sum) in Caribbean corals [32]. Isoprenoid hydrocarbons: pristane (Pr), phytane (Ph) and squalene (Sq). na – no analysis.

Order and species	*n*-Alkanes															Isoprenoids		
	C16	C17	C18	C19	C20	C21	C22	C23	C24	C25	C26	C27	C28	C29	C30	Pr	Ph	Sq
Pocilloporidae																		
Madracis decactis	1.5	3.0	1.3	0.7	1.3	0.4	1.2	3.8	8.3	11.6	9.8	5.8	3.1	2.2	0.8	-	0.5	5.5
Madracis decactis	3.9	6.2	5.2	4.1	1.8	0.8	0.8	1.5	0.5	1.2	0.5	0.6	-	-	-	4.6	3.2	0.4
Madracis decactis	1.2	3.2	2.0	2.2	0.6	0.4	0.4	0.7	-	-	0.9	0.8	1.3	4.7	3.9		1.2	0.7
Madracis decactis	5.9	10.3	4.6	12.8	0.9	0.4	0.1	0.2	0.6	1.1	0.5	-	0.1	-	0.1	8.0	4.7	6.3
Madracis mirabilis	2.2	13.0	4.2	3.7	1.6	0.9	1.5	2.0	3.1	4.5	8.8	1.8	3.5	1.1	1.3	4.2	2.1	1.2
Agariciidae																		
Agaricia agaricites	4.8	5.5	3.6	2.7	1.0	0.6	0.6	0.6	0.6	1.6	0.7	0.8	0.1	1.1	0.5	3.0	1.7	na
Helioseris cucullata	8.8	12.7	8.3	5.5	2.2	1.3	1.6	1.8	2.3	3.5	2.3	1.9	2.2	2.1	1.4	6.1	3.7	na
Poritidae																		
Porites astreoides	3.1	6.3	3.6	-	-	-	-	-	-	80.0	-	-	-	-	-	4.1	-	6.2
Porites porites	0.4	3.1	2.6	2.6	0.4	-	-	1.6	-	54.7	-	-	2.5	-	1.7	2.8	1.6	1.0
Porites divaricata	1.2	4.8	2.6	2.3	2.4	-	0.4	0	0.3	-	0.8	-	1.2	12.5	1.6	2.2	1.4	-
Porites divaricata	2.0	7.2	2.3	3.1	-	0.4	2.0	3.4	4.3	4.8	4.7	4.2	6.2	5.8	4.4	3.4	2.4	0.4
Porites divaricata	4.8	15.2	6.0	3.7	3.2	0.4	1.8	0.3	1.8	-	2.1	-	1.1	7.4	5.1	5.4	3.4	1.9
Porites furcata	1.9	4.4	1.3	0.9	1.5	0.8	2.1	5.4	7.5	8.6	6.9	6.3	3.5	2.2	1.2	0.9	0.5	10.6
Porites furcata	8.8	14.4	8.5	-	-	-	-	-	-	61.8	-	-	-	-	-	-	-	2.2
Faviidae																		
Manicina areolata	-	6.6	4.2	3.9	-	-	-	2.2	3.4	40.7	4.4	4.0	2.8	3.0	4.6	4.4	2.3	3.5
Montastrea annularis	5.3	5.9	4.9	3.0	3.2	0.8	0.9	0.4	1.2	1.5	1.6	0.6	1.0	2.6	-	4.1	2.3	0.7
Solenastrea hyades	0.4	1.0	0.4	0.2	0.2	-	0.2		1.2	1.4	10.8	0.8	-	-	-	5.5	4.0	1.4
Solenastrea hyades	3.0	3.4	2.2	2.1	1.0	0.4	1.5	0.8	2.0	3.6	1.7	-		4.5	4.4	1.8	1.4	3.0
Oculinidae																		
Oculina diffusa	-	5.0	0.7	6.0	3.8	1.2	1.8	0.5	1.4	-	1.1	1.0	0.6	-	2.5	3.9	-	1.4

Meandrinidae																				
Dichocoenia stokesii			2.4	1.8	2.5	1.2	na	na	na	na	na	na	na	na	na	na	na	6.6	4.3	1.1
Mussidae																				
Mussa angulosa			1.5	25.3	2.5	4.0	1.1	0.9	0.7	0.7	0.9	0.3	0.5	-	0.3	-	0.1	0.9	1.2	na
Scolymia lacera			0.2	0.7	-	0.2	0.6	-	-	-	-	-	-	-	-	-	-	-	-	0.2
Isophyllia sinuosa			0.3	2.9	0.5	0.5	0.2	0.1	0.1	0.1	0.2	-	-	-	-	-	-	0.2	0.2	na
Isophyllastrea rigida			1.0	13.7	3.7	5.8	1.3	0.6	0.6	0.7	1.2	2.3	0.5	-	-	-	0.6	1.9	2.2	na
Mycetophyllia ferox			0.2	0.9	2.0	2.2	1.0	0.8	0.9	1.6	1.3	4.4	1.0	4.3	0.7	1.7	0.2	-	0.8	na
Milleporidae																				
Millepora alcicornis			2.2	3.5	2.1	1.6	1.3	0.2	0.8	0.3	1.0	1.4	1.2	2.0	0.6	2.1	1.9	2.2	0.6	-
Millepora alcicornis			3.1	3.0	2.2	1.0	1.2	0.2	2.0	0.5	2.2	-	1.5	-	-	3.2	1.9	2.4	1.3	27.4
Millepora alcicornis			2.5	7.1	2.2	1.5	1.1	0.4	1.1	0.8	1.6	1.3	1.6	2.1	1.7	2.4	2.9	1.1	1.0	0.9

Table S4. Composition of coral wax FA (% of total FA), collected at the Great Barrier Reef (Australia) [33]. Numeration of the coral species is as in the Table 1.

No	Fatty acids										
	14:0	16:1	16:0	18:4+ 18:5	18:2+ 18:3	18:1	18:0	20:U	20:0	22:U	22:0
1	1.1	1.3	46.8	7.0	0.8	7.4	7.2	8.3	-	9.9	-
2	-	-	37.4	8.2	-	2.5	4.3	6.5	-	41.0	-
3	4.7	2.4	78.3	3.6	1.4	2.5	3.8	1.1	0.4	1.0	6.3
4	-	-	50.6	-	-	4.8	6.6	1.5	-	36.6	-
5	-	-	77.2	3.8	4.4	-	14.6	-	-	-	-
6	1.8	2.0	66.1	0.9	0.6	6.5	13.7	3.9	1.1	3.1	0.2
7	-	14.3	17.3	0.6	-	62.2	2.8	2.8	0.6	-	-
8	-	15.0	15.8	0.4	-	58.2	2.5	6.0	-	-	-
9	7.6	1.4	75.2	0.4	0.4	5.0	7.4	0.6	1.0	0.7	0.2
10	4.7	1.3	31.7	0.4	-	57.0	4.3	0.5	0.2	-	-
11	8.4	3.0	63.4	3.7	1.2	7.1	7.0	2.4	0.7	3.0	0.2
12	-	-	72.6	0.8	2.7	12.6	7.2	0.3	1.2	2.0	0.6
13	13.0	2.5	59.5	2.1	0.3	20.3	2.4	-	-	-	-
14	9.0	5.0	79.3	1.6	2.6	-	2.4	-	-	-	-
15	1.1	0.9	61.7	1.6	-	19.4	6.9	1.5	-	7.0	-
16	-	2.1	57.4	4.2	8.6	-	12.7	4.9	0.4	9.8	-
17	-	-	1.8	2.2	-	2.4	14.7	18.6	-	60.2	-
18	0.5	4.1	55.0	5.0	1.2	14.1	7.9	5.7	0.3	6.2	-
19	2.2	2.2	68.2	3.9	1.2	6.9	8.2	1.9	0.7	4.5	0.1
20	-	3.4	76.9	7.8	-	2.1	8.6	-	-	1.1	-
21	3.7	1.1	62.7	2.9	0.3	5.5	18.3	3.4	1.3	0.8	-
22	7.1	5.1	68.5	6.8	1.1	7.0	4.1	1.1	0.1	0.1	-

23	7.1	3.9	58.0	5.3	1.9	7.8	8.0	2.5	0.6	4.0	0.9
24	1.8	3.1	59.7	1.3	-	30.0	4.1	-	-	-	-
25	9.1	9.6	65.9	2.2	1.0	9.6	2.6	-	-	-	-
26	1.2	6.9	72.3	4.7	1.7	6.1	3.2	0.1	-	3.7	-
27	1.4	4.7	72.7	1.8	1.8	4.1	3.9	0.8	0.5	1.8	6.6
28	5.3	2.6	78.6	0.8	-	4.1	7.6	-	1.0	-	-
29	8.8	3.5	70.3	5.0	0.1	6.4	5.9	-	-	-	-
30	1.2	1.8	65.6	6.5	1.1	5.5	11.1	2.3	0.6	3.2	-
31	2.5	8.9	58.3	8.6	1.3	4.2	6.0	1.7	-	-	8.5
32	5.4	7.2	54.8	1.0	0.6	12.0	6.2	3.1	-	9.9	-
33	0.7	5.9	38.6	7.8	2.3	8.6	2.2	5.9	-	28.0	-
34	3.5	9.3	55.7	0.7	2.2	15.3	5.5	3.5	0.2	3.0	1.0
35	6.9	-	79.3	-	-	11.6	2.6	-	-	-	-
36	6.3	1.2	66.0	4.0	1.3	5.1	9.2	6.3	0.3	0.4	-
37	-	-	22.3	6.6	1.2	0.3	6.7	8.3	2.3	29.2	23.21
38	2.4	1.6	50.4	6.8	3.4	8.4	7.9	3.5	0.8	8.1	6.8

Table S5. Fatty acid composition (% of total) of wax esters of cnidarians (Okinawa Is., Japan) [36].

Fatty acids	*Pocillopora damicornis*	*Pocillopora verrucosa*	*Stylophora pistillata*	*Montipora aequituberculata*	*Acropora microphthalma*	*Porites lutea*	*Porites cylindrica*	*Fungia fungites*	*Galaxea fascicularis*	*Goniastrea aspera*	*Oulastrea crispata*	*Tubastrea* sp.	*Lobophytum crassum*	*Millepora murrayi*
14:0	4.2	3.9	7.1	2.0	0.9	1.8	6.7	2.4	6.0	1.5	3.2	0.3	3.1	0.0
16:0	49.2	62.1	53.1	82.7	73.2	62.4	70.6	51.5	65.4	45.5	21.7	24.5	76.7	30.9
16:1n-7	7.8	6.4	5.6	0.0	4.4	2.2	1.0	3.5	7.5	0.0	32.7	8.1	4.4	0.0
18:0	7.3	5.5	5.3	7.0	4.8	6.5	11.7	8.4	5.0	9.4	2.0	6.3	8.2	51.0
18:1n-9	16.2	16.0	17.6	6.2	3.6	22.6	11.6	10.5	9.2	15.6	37.3	41.8	2.5	8.9
18:2n-6	2.3	1.9	1.8	0.3	0.0	0.8	0.5	1.0	1.5	2.8	0.6	0.0	3.2	5.5
18:3n-6	1.5	0.0	0.0	0.0	0.0	0.0	0.0	10.6	3.0	8.9	0.0	2.1	0.0	0.0
18:4n-3	1.2	1.0	0.0	0.4	1.3	0.0	0.0	0.8	0.0	1.0	0.0	0.0	0.0	1.5
20:0	0.7	0.6	0.0	0.2	0.0	0.4	0.5	0.5	0.1	0.6	0.1	0.0	1.2	2.0
20:1	3.2	1.8	4.0	0.2	2.7	1.8	1.4	0.4	0.0	3.4	1.7	5.4	0.0	0.0
20:2	0.5	0.0	1.1	0.0	0.7	0.6	0.5	0.0	0.0	0.5	0.0	0.0	0.0	0.0
20:3n-6	1.8	0.0	1.1	0.3	0.0	0.3	0.0	1.4	0.0	2.7	0.0	0.0	0.0	0.0
20:4n-6	0.4	0.1	0.5	0.0	0.0	0.6	0.2	0.2	0.9	0.5	0.0	0.0	0.0	0.0
20:5n-3	0.2	0.1	0.0	0.0	0.0	0.0	0.2	0.3	0.0	0.0	0.0	1.7	0.0	0.0
22:6n-3	1.4	0.0	1.0	0.0	2.2	0.0	0.0	0.0	0.0	0.0	0.0	0.0	0.0	0.0

Table S6. Fatty acid composition (% of total) of triacylglycerols from corals collected at the Great Barrier Reef (Australia) [33]. Numeration of the coral species as in the Table 1.

No.	Fatty acids										
	14:0	16:1	16:0	18:4+ 18:5	18:2+ 18:3	18:1	18:0	20:U	20:0	22:U	22:0
1	6.0	5.5	44.9	4.9	0.9	11.6	6.0	10.4	0.6	8.3	1.0
2	1.0	4.1	62.7	8.9	0.6	5.6	6.7	-	0.5	9.7	-
3	9.3	2.1	63.1	9.2	1.4	2.9	3.8	3.6	0.4	4.4	-
4	3.2	4.3	63.5	12.4	2.9	3.7	3.7	3.8	-	2.4	-
5	1.5	-	59.5	11.0	1.2	5.5	14.9	4.9	1.6	-	-
6	2.4	2.3	65.5	3.7	0.8	7.9	8.1	4.5	1.4	3.3	-
7	1.4	1.9	37.8	0.4	0.9	17.8	18.7	2.1	0.2	16.8	2.2
8	1.3	4.5	33.9	0.3	2.3	17.2	11.0	2.0	0.9	20.0	1.9
9	3.5	-	57.8	7.7	1.9	10.7	6.7	9.5	0.3	2.0	-
10	7.1	8.7	61.8	5.4	-	7.6	8.0	1.4	-	-	-
11	12.6	4.2	66.9	4.4	2.8	2.7	5.3	1.1	-	0.1	-
12	5.7	5.5	76.8	3.0	4.6	-	4.4	-	-	-	-
13	9.6	4.4	68.8	5.0	7.5	-	4.7	-	-	-	-
14	4.4	4.6	66.1	10.5	0.3	4.5	5.0	0.2	-	4.4	-
15	1.7	2.4	61.7	6.2	0.5	17.0	8.5	1.8	-	0.3	-
16	1.0	3.2	58.0	2.3	2.0	5.8	9.4	6.8	-	10.0	3.3
17	2.0	3.5	54.8	6.5	-	6.3	11.2	8.9	0.6	6.4	-
18	1.7	2.1	59.7	4.5	0.4	9.0	12.0	6.1	0.5	4.2	-
19	2.5	3.8	58.0	6.4	0.9	7.5	6.2	6.4	0.9	7.3	0.2
20	1.5	-	68.1	9.8	0.4	4.2	9.5	4.2	-	2.4	-
21	6.3	4.2	73.1	5.1	4.5	-	6.8	-	-	-	-
22	1.7	3.6	57.2	12.1	1.0	6.9	5.1	5.7	0.4	6.2	-
23	6.1	1.8	60.6	7.9	1.2	6.9	6.8	4.0	0.6	4.2	-
24	0.6	2.3	39.7	7.8	0.4	41.6	6.7	0.9	-	-	-
25	0.3	1.4	35.4	1.0	-	53.8	5.5	1.4	-	1.1	-
26	1.3	6.4	50.2	17.3	4.0	5.8	4.7	4.4	0.3	5.5	0.1
27	1.4	5.8	64.9	7.7	2.7	5.0	4.3	3.1	0.3	4.7	0.1
28	4.6	7.2	73.5	2.8	-	6.2	5.8	-	-	-	-
29	2.8	2.4	41.0	7.4	15.4	0.7	8.5	10.0	0.4	11.4	-
30	2.3	3.0	60.2	7.1	4.5	1.9	9.6	6.5	0.7	4.0	-
31	3.5	5.3	71.4	5.6	1.2	1.8	6.1	3.0	0.2	2.0	-
32	6.6	5.2	43.4	2.0	1.6	10.2	7.4	7.0	0.3	16.4	-
33	4.3	2.6	49.5	6.3	-	4.3	6.5	4.7	0.3	21.3	0.2
34	6.0	4.8	48.0	1.5	1.2	9.9	4.6	7.9	0.3	15.9	-
35	1.9	0.6	53.5	2.7	0.5	26.7	8.2	1.1	-	4.9	-
36	8.0	2.9	66.7	6.2	1.0	6.9	6.7	1.4	-	0.3	-
37	-	5.4	41.3	7.0	-	5.8	13.4	-	0.9	12.7	0.2

38	3.2	2.1	57.7	7.0	2.0	7.9	6.8	6.8	-	6.5	-

Table S7. Fatty acid composition (% of total) of triacylglycerols of cnidarians, Okinawa Is., Japan [36].

Fatty acids	*Pocillopora damicornis*	*Pocillopora verrucosa*	*Stylophora pistillata*	*Montipora aequituberculata*	*Acropora microphthalma*	*Porites lutea*	*Porites cylindrica*	*Fungia fungites*	*Galaxea fascicularis*	*Goniastrea aspera*	*Oulastrea crispata*	*Tubastrea* sp.	*Lobophytum crassum*	*Millepora murrayi*
14:0	5.6	7.9	6.3	2.2	4.3	0.9	1.2	1.7	3.9	1.2	8.6	4.2	1.4	8.4
16:0	51.3	63.2	57.7	79.8	57.9	49.8	61.8	64.5	55.6	66.3	50.2	48.1	67.0	48.9
16:1n-7	3.0	2.7	4.1	2.4	3.3	0.9	1.0	1.7	3.0	1.1	5.4	4.4	5.2	0.2
18:0	7.4	6.6	5.0	5.8	8.7	8.1	9.0	8.7	5.2	10.3	5.3	15.7	8.8	32.7
18:1n-9	6.0	6.2	13.5	3.0	6.6	21.6	17.9	7.0	6.3	4.6	16.1	5.1	3.4	3.4
18:2n-6	1.2	0.6	1.0	0.4	0.8	0.7	1.0	0.8	1.6	0.6	0.0	0.0	0.7	0.6
18:3n-6	3.0	1.0	0.6	0.0	6.2	3.5	1.2	5.9	13.2	2.4	0.0	0.0	0.0	0.5
18:4n-3	0.9	0.0	0.3	0.7	0.0	0.1	1.1	1.2	2.2	0.4	0.0	0.0	0.0	0.7
20:0	1.8	0.8	0.4	0.3	1.3	0.3	0.2	0.5	0.3	0.8	0.5	0.8	0.6	2.0
20:1	4.3	4.0	2.0	1.1	2.8	1.3	0.6	0.9	0.1	1.3	2.2	2.5	0.0	0.0
20:2	1.5	0.6	0.9	0.6	0.4	0.7	0.5	0.0	0.3	0.8	1.8	1.1	0.0	0.0
20:3n-6	2.5	2.3	1.6	0.0	1.7	0.5	0.2	1.5	2.7	0.8	0.0	0.0	0.0	0.0
20:4n-3	2.7	0.0	1.4	0.0	0.4	0.3	0.2	0.2	0.2	0.1	0.0	0.0	0.0	0.0
20:4n-6	0.1	0.0	0.1	0.0	0.4	1.6	0.8	1.0	0.9	1.3	0.3	0.4	0.0	0.0
20:5n-3	0.4	0.0	0.7	0.0	1.2	3.3	1.5	0.0	0.5	0.1	0.4	1.1	0.3	0.0
22:0	0.2	1.2	0.0	0.0	0.0	0.2	0.1	0.4	0.1	0.0	0.1	0.0	0.2	0.0
22:1	0.7	0.0	0.5	0.0	0.3	0.3	0.1	0.4	0.1	1.2	1.5	0.8	0.0	0.0
22:4n-3	0.4	0.0	0.1	0.0	0.2	0.7	0.2	0.0	0.2	0.3	0.4	0.2	0.0	0.0
22:5n-3	0.6	0.4	0.8	0.4	0.7	1.0	0.4	0.2	0.2	0.3	0.0	1.9	0.2	0.4
22:6n-3	3.9	0.0	2.0	0.0	1.4	3.2	0.8	0.0	1.5	0.3	0.4	0.2	0.0	0.0

Table S8. FA composition (% of total) of lipids of the soft coral *Sinularia* sp. from the coast waters of Vietnam [79].

Fatty acids	Triacylglycerols	Monoalkyldiacylglycerols	Lipids		
			Total	Neutral	Polar
14:0	1.3	1.6	2.3	1.9	2.4
16:0	47.4	42.8	47.0	38.3	24.3
16:1n-9	0.5	0.6	0.4	0.5	0.3
16:1n-7	2.6	3.0	3.0	3.8	2.1
16:2n-7	6.3	6.6	5.2	7.6	3.3
16:3n-4	1.0	1.3	1.7	2.0	6.3
16:4n-1	-	0.7	0.6	0.4	2.3
18:0	10.2	12.5	10.7	8.2	7.2

18:1n-9	7.3	4.1	4.8	5.9	3.8
18:1n-7	0.9	0.9	0.8	0.9	0.8
18:2n-9	0.6	0.9	0.6	0.8	-
18:2n-7	2.0	4.1	2.8	4.2	0.7
18:2n-6	11.5	0.9	4.0	6.2	1.9
18:3n-6	0.9	2.7	1.3	2.1	-
18:3n-3	0.9	0.4	0.5	0.8	0.6
18:4n-3	0.8	1.2	1.4	1.4	14.1
20:0	0.7	1.0	0.9	0.6	0.3
20:3n-6	-	0.7	0.3	0.4	0.2
20:4n-6	0.8	6.3	4.2	5.2	9.0
20:4n-3	0.5	1.8	0.8	1.3	0.2
20:5n-3	0.4	0.8	0.5	0.5	3.9
22:0	0.5	0.8	0.7	0.4	0.2
22:4n-6	-	-	0.1	-	0.6
22:6n-3	1.3	1.2	1.0	1.5	2.3
24:5n-6	0.1	0.2	1.7	2.0	9.5
24:6n-3	0.1	0.1	0.7	1.0	1.0

Table S9. The FA composition (%) of the total lipids of Caribbean corals [32].

Family, species	12:0	14:0	16:0	16:1	18:0	18:1	20:0	20:1	20:5	22:0	22:1	22:5	22:6	24:0	24:1
Pocilloporidae															
Madracis decactis	0.5	4.7	40.4	tr	8.0	13.4	3.9	tr	0.7	tr	3.1	5.0	20.3	-	-
Madracis decactis	0.3	4.5	38.3	12.6	11.2	9.6	-	-	3.5	-	8.0	2.4	2.8	-	6.5
Madracis decactis	0.2	22.3	39.6	11.7	8.4	10.6	0.5	1.2	1.0	-	1.7	0.9	1.8		-
Madracis decactis	0.6	9.1	49.6	10.4	7.1	20.0	1.0	2.2	-	-	-	-	-	-	-
Madracis mirabilis	0.3	7.7	66.8	-	5.2	16.0	2.6	-	-	-	-		-	1.4	-
Acroporidae															
Acropra palmata	0.3	6.3	60.7	tr	11.2	9.8	2.2	3.6	0.7	0.6	1.5	1.6	tr	tr	1.5
Acropra palmata	0.1	5.5	80.3	0.3	2.9	9.1	0.5	1.3	-	-	-	-	-	-	-
Acropra cervicornis	0.2	6.6	56.3	6.6	7.6	10.2	2.4	3.0	0.8	0.2	1.3	1.7	1.2	tr	1.2
Acropra cervicornis	0.3	5.5	64.2	5.6	5.0	9.3	1.5	2.8	0.9	0.4	0.9	1.5	tr	tr	1.2
Acropra cervicornis	0.1	12.3	78.1	2.5	3.2	2.5	-	tr	0.5	0.4	0.5	-	-	-	-
Acropra prolifera	0.1	6.5	67.4	-	11.3	11.3	0.4	3.0	-	-	-	-	-	-	-
Agariciidae															
Agaricia agaricites	0.2	2.7	55.1	tr	20.0	tr	5.5	-	1.9	tr	4.5	2.2	7.8	-	-
Agaricia agaricites	0.6	6.8	64.1	7.6	7.5	5.0	1.8	-	1.5	0.2	1.7	-	2.0	1.2	-
Helioseris cucullata	0.5	5.9	52.0	10.6	3.4	11.2	6.0	-	3.0	0.3	2.3	-	4.9	-	-
Siderastreidae															
Siderastrea siderea	0.3	3.5	32.5	tr	13.2	18.6	0.6	7.0	4.3	tr	15.2	3.6	1.2	-	-
Poritidae															
Porites astreoides	0.6	3.2	30.0	tr	11.4	12.3	7.6	tr	1.4	-	14.6	1.7	7.8	-	9.5
Porites astreoides	0.2	5.2	70.6	-	6.8	15.3	-	1.8	-	-	0.1	-	-	-	-
Porites porites	1.4	5.8	48.7	tr	9.0	12.2	2.6	4.4	3.1	-	6.9	1.9	3.9	-	-
Porites porites	0.1	7.0	64.4	-	4.3	12.3	3.7	3.1	-	-	4.6	-	-	0.6	-
Porites divaricata	0.1	1.6	23.1	6.3	10.7	8.2	8.3	tr	2.0	tr	6.4	3.0	30.3	-	-
Porites divaricata	0.1	4.7	48.9	-	16.8	8.2	5.3	5.2	-	-	4.3	2.1	4.7	-	-
Porites divaricata	0.2	3.8	59.8	5.2	10.8	10.6	1.6	2.9	-	-	1.4	-	-	-	-
Porites furcata	0.3	3.8	43.8	15.1	5.4	9.8	2.1	2.4	2.2	tr	8.1	2.3	4.7	-	-
Porites furcata	0.1	3.4	34.5	tr	6.2	9.6	6.8	3.8	6.9	tr	6.0	4.4	18.3	-	-
Porites furcata	0.1	5.9	64.6	-	11.8	13.8	1.3	2.5	-	-	-	-	-	-	-
Faviidae															
Manicina areolata	0.1	3.4	36.5	4.4	12.7	25.4	3.0	9.8	-	-	4.0	-	-	1.4	-
Colpophyllia natans	0.1	4.3	35.8	tr	19.1	17.7	4.7	6.1	0.5	0.9	6.2	1.2	1.6	-	1.6
Colpophyllia breviserialis	0.1	3.9	36.3	tr	21.6	15.6	4.4	5.1	0.7	0.8	6.5	0.9	1.4	-	2.1
Cladocora arbuscula	0.1	8.6	36.3	16.4	7.9	14.7	2.1	3.8	1.1	1.0	3.6	-	1.8	2.6	-
Montastrea annularis	tr	2.0	58.1	tr	7.2	11.6	6.2	tr	-	3.3	5.3	-	-	5.9	-
Montastrea cavernosa	0.1	3.8	56.0	-	14.1	12.3	2.1	3.1	1.4	tr	3.0	0.7	2.7	-	1.1
Solenastrea hyades		1.5	73.0	-	7.3	5.4	4.8	-	0.3	-	1.8	2.5	2.2	-	1.0
Solenastrea hyades	0.2	1.6	76.3	-	9.5	6.4	3.1	-	0.2	-	1.0	0.5	0.4	-	0.8

Oculinidae															
Oculina diffusa	0.1	5.6	66.3	1.5	5.3	18.6	0.5	0.4	0.5	-	0.7	-	-	-	0.5
Meandrinidae															
Dichocoenia stokesii	0.1	0.8	19.8	tr	11.3	9.7	7.1	tr	2.6	-	8.1	4.3	29.2	-	6.9
Mussidae															
Mussa angulosa	0.1	9.4	-	67.3	4.8	14.7	1.2	2.2	0.1	tr	-	-	0.1	-	0.1
Scolymia lacera	0.1	3.7	29.2	tr	8.0	20.7	6.9	7.7	3.3	-	7.8	2.1	10.9	-	-
Isophyllia sinuosa	tr	4.1	46.0	8.8	9.1	17.2	3.6	3.5	0.4	0.3	5.8	-	0.3	0.7	-
Isophyllastrea rigida	0.2	4.6	14.0	33.0	11.5	17.5	3.2	4.7	1.0	0.4	6.7	-	1.6	1.7	-
Mycetophyllia ferox	0.2	5.1	75.1	-	6.5	11.2	0.5	tr	0.3	0.1	1.0	-	-	tr	-
Dendrophylliidae	:														
Balanophyllia floridana	0.4	5.9	28.0	11.1	14.3	15.5	3.9	7.0	3.4	tr	6.6	tr	4.0	tr	tr

Table S10. The FA composition (%) of the total lipids of Jamaican hard corals *Montastrea annularis* and *Stephanocoenia michelinii* versus the depth of habitat [97,98].

FA	*Montastrea annularis*						*Stephanocoenia michelinii*						
	Depth, m												
	3	6	9	12	18	24	3	6	9	12	14	18	24
14:0	2.5	3.8	1.5	2.5	0.9	1.8	0.3	1.9	0.6	2.6	1.0	1.5	1.0
16:0	65.4	62.5	61.0	66.4	72.3	70.3	6.9	43.2	54.9	68.3	29.3	50.7	52.6
16:1	4.2	5.1	2.0	4.8	2.6	0	0.5	3.0	0	2.9	1.8	2.1	2.1
18:0	12.2	8.2	8.3	9.4	12.1	18.8	2.6	9.7	19.1	11.6	7.6	13.9	15.3
18:1	8.8	12.4	11.7	10.3	7.8	7.0	2.1	7.8	15.3	8.4	18.9	17.4	15.8
18:2	1.1	1.6	2.0	1.1	0.6	0.6	0.4	2.5	1.1	2.1	1.5	3.0	1.9
20:0	0.1	0.5	0.1	0.5	1.0	0	0.6	3.7	4.4	2.0	2.0	5.4	4.5
20:1	0.1	0	0.6	2.0	2.8	1.5	0.5	0	4.8	2.1	5.9	5.9	4.0
22:6	0	2.2	1.4	0	0	0	82.8	16.4	0	0	15.0	0	0

Table S11. The FA composition (%) of the total lipids of 8 species of hard corals, collected at the Great Barrier Reef (Australia) [99].

FA	Florida, Middle Ground, July, 25-30 m						Grand Cayman, March, 2-5 m	
	Porites furcata	*Porites devaricata*	*Cladocora arbuscula*	*Dichocoenia stokesii*	*Madracis decactis*	*Millepora alcicornis*	*Porites furcata*	*Acropora palmata*
12:0	0	0.1	0.1	0.1	0.5	0.1	0.3	0.3
14:0	3.4	1.6	8.6	0.8	4.7	3.4	3.8	6.3
16:0	34.5	23.1	36.3	19.8	40.4	42.1	43.8	60.7
18:0	6.2	10.7	7.9	11.3	8.0	5.4	5.4	11.2
20:0	6.8	8.3	2.1	7.1	3.9	4.7	2.1	2.2
22:0	tr	tr	1.0	0	tr	1.0	tr	0.6
24:0	0	0	2.6	0	0	tr	0	tr
16:1	tr	6.3	16.4	tr	tr	tr	15.1	tr

18:1	9.6	8.2	14.7	9.7	13.4	11.1	9.8	9.8
20:1	3.8	tr	3.8	tr	tr	tr	2.4	3.6
22:1	6.0	6.4	3.6	8.1	3.1	1.0	8.1	1.5
24:1	0	0	0	6.9	0	2.1	0	1.5
20:5n-3	6.9	2.0	1.1	2.6	0.7	1.2	2.2	0.7
22:5	4.4	3.0	0	4.3	5.0	4.5	2.3	1.6
22:6n-3	18.3	30.3	1.8	29.2	20.3	23.5	4.7	tr

Table S12. The FA composition (%) of the hard corals total lipids of Vietnam (B) and Seychelles (C) [100].

Fatty acid	Coral family																	
	Acroporidae					Pocilloporidae								Poritidae			Dendrophyllidae	
	Acropora nasuta	*Acropora nasuta*	*Acropora millepora*	*Acropora millepora*	*Acropora florida*	*Seriatopora caliendrum*	*Stylophora pistillata*	*Stylophora pistillata*	*Stylophora pistillata*	*Stylophora pistillata*	*Pocillopora damicornis*	*Pocillopora damicornis*	*Pocillopora verrucosa*	*Porites lutea*	*Goniopora* sp. I	*Goniopora* sp. II	*Tubastrea coccinea*	*Tubastrea micrantha*
	B	B	B	B	B	C	C	C	B	B	B	B	B	B	B	B	C	C
14:0	5.4	2.5	1.2	3.6	2.7	1.8	2.0	3.3	4.0	3.5	3.9	3.6	5.4	5.4	2.2	2.0	0.9	0.5
16:0	42.5	38.6	24.5	41.3	33.1	24.0	21.8	18.4	38.6	41.0	37.2	44.5	41.7	49.1	17.2	15.0	7.2	6.4
16:1n-7	0.8	1.0	1.0	2.6	1.2	2.1	2.9	4.1	3.2	3.0	2.2	3.1	2.3	1.9	3.5	2.3	5.9	3.7
16:2	-	1.8	-	0.7	-	0.9	-	1.3	0.6	0.5	0.6	0.3	0.5	0.7	0.5	1.2	3.1	2.9
18:0	6.9	7.3	9.3	8.7	9.0	6.5	4.6	8.6	11.4	10.6	11.4	10.2	8.0	7.2	5.7	6.8	4.2	6.7
18:1n-9	3.7	8.0	2.2	6.5	3.2	13.3	14.4	7.8	3.3	5.5	3.3	4.9	7.0	3.8	11.7	6.6	23.3	26.4
18:2n-6	0.7	2.1	1.1	1.7	1.3	1.7	1.7	0.8	1.7	1.9	1.7	1.8	1.2	1.0	2.2	1.6	2.0	1.8
18:3n-6	9.7	5.8	9.5	5.0	8.2	2.7	3.1	1.7	5.6	5.4	5.6	4.1	2.6	9.7	4.5	6.2	0.4	0.3
18:4n-3	4.9	2.6	6.6	1.1	5.1	1.7	1.3	1.9	2.0	1.4	2.2	0.8	3.3	2.9	2.3	4.3	0.7	0.8
20:0	-	0.5	-	1.3	-	-	-	0.8	1.7	1.6	1.7	1.5	1.3	1.0	-	-	0.4	1.0
20:1	1.3	0.7	1.1	1.6	1.0	0.9	0.7	0.7	0.7	1.4	3.5	1.1	2.6	0.9	5.9	2.9	3.0	3.2
20:2n-6	0.2	2.0	0.2	0.7	0.5	2.7	3.6	1.0	0.6	0.8	0.6	0.6	0.7	-	1.7	1.4	0.9	1.6
20:3n-6	2.3	1.9	2.4	2.0	0.3	11.3	12.3	5.9	6.6	7.2	4.1	7.6	3.2	1.6	3.6	2.9	0.8	0.5
20:4n-6	3.2	7.1	7.2	8.0	11.0	4.8	4.3	7.6	3.1	1.7	2.1	2.0	1.8	2.3	13.3	21.9	7.8	6.6
20:4n-3	0.3	0.2	-	0.5	-	1.3	1.7	0.9	0.4	0.4	1.3	0.4	1.0	0.3	0.7	1.1	0.9	1
20:5n-3	4.5	0.8	10.4	1.6	6.9	2.6	2.0	7.7	2.6	1.4	1.8	1.4	3.2	3.3	4.1	4.6	14.9	10.9
22:3n-6	-	-	-	0.6	0.3		1.9	0.9	0.5	0.6	0.3	0.6	0.3	-	0.4	0.3	0.5	0.3

22:4n-6	2.4	4.3	6.0	1.0	6.3	1.5	1.8	3.8	1.7	1.0	1.3	0.9	1.3	1.4	3.3	6.0	4.7	5.5
22:5n-6	0.3	-	0.6	-	1.3	-	-	-	-	-	-	-	-	-	-	-	-	-
22:5n-3	1.3	0.9	3.0	0.5	1.2	1.2	1.3	4.5	0.7	4.5	0.6	0.4	0.7	0.8	1.0	0.8	16.4	17.3
22:6n-3	8.8	10.8	12.6	10.4	6.7	16.9	16.4	14.4	10.1	8.8	14.0	9.5	10.4	5.3	15.7	11.5	1.4	1.3

Table S13. The FA composition (%) of total lipids of 5 hard coral species of the Caribbean Islands and the Red Sea [35] and cultivated for one month colony of *Galaxea fascicularis* [101].

FA	Caribbean Islands		Red Sea			*Galaxea fascicularis*
	Porites porites	*Montastrea annularis*	*Pocillopora verrucosa*	*Stylophora pistillata*	*Goniastrea retiformis*	
14:0	1.2	1.1	2.3	1.6	2.4	1.9
16:0	30.0	58.6	36.8	27.3	49.5	17.3
16:ln-9	1.1		0.1	0.3		-
16:ln-7	0.5	1.9	5.1	3.8	2.7	2.3
16:2	-	-	-	-	0.3	-
17:0	-	-	0.1	-	0.2	0.4
18:0	9.5	12.4	9.9	7.1	16.8	6.8
18:ln-9	5.9	7.1	8.3	10.7	4.7	1.3
18:ln-7	0.5	1.4	2.0	3.2	2.8	-
18:2n-6	4.0	1.2	2.2	1.3	1.3	0.6
19:0	5.1	3.3	5.5	2.9	3.7	-
19:1	0.2	0.2	0.1	-	0.2	-
18:3n-3	-	-	0.1	0.1	-	5.8
18:4n-3	1.4	0.2	1.9	2.6	0.7	7.3
20:0	0.7	0.8	1.9	0.5	1.1	-
20:ln-ll	-	-	0.1	0.4	-	0.8
20:ln-9	0.9	1.9	4.8	2.6	1.4	0.3
20:ln-7	1.4	0.2	0.5	0.4	0.2	-
20:2n-6	2.4	0.3	0.8	0.6	0.3	-
20:3n-6	1.3	1.4	6.5	6.9	1.3	1.2
20:4n-6	9.1	2.2	1.5	4.9	3.1	14.5
20:4n-3	-	-	1.3	2.3	0.3	0.1
20:5n-3	5.0	0.5	0.6	1.5	0.6	10.4
22:0	0.3		0.2	0.2	0.3	-
22:ln-9	0.4	0.3	0.3	0.3	0.2	-
22:3n-6	-	-	0.5	0.7	-	-
22:3n-3	-	-	-	-	-	10.1
22:5n-6	5.2	0.9	1.3	2.8	1.6	-
22:5n-3	2.1	0.3	0.3	0.6	-	1.3

22:6n-3	6.7	2.9	3.6	12.0	2.1	9.6
24:ln-9	1.3	-	-	0.6	-	-

Table S14. The total lipids FA composition (% of total, mean ± SD, *n* = 3) of the Acroporidae family corals collected at Vietnam coast in spring [103]. i = *iso*-, and ai = *anteiso*-FA

FA	*Acropora cerealis*	*Acropora formosa*	*Acropora gemmifera*	*Acropora palifera*	*Acropora sp.*	*Acropora nobilis*
12:0	0.5±0.2	0.2±0.0	0.6±0.4	0.7±0.2	0.2±0.1	0.1±0.1
14:0	3.2±0.1	2.9±0.4	3.6±0.1	2.3±0.1	4.4±0.2	5.9±0.3
14:1	0.2±0.1	0.2±0.1	0.3±0.1	0.2±0.1	0.1±0.0	0.2±0.1
i-15:0	0.2±0.0	0.2±0.0	-	-	0.3±0.1	0.4±0.0
15:0	0.1±0.0	0.2±0.0	0.2±0.0	0.1±0.0	0.1±0.0	0.3±0.1
15:1	0.3±0.2	0.3±0.1	0.1±0.0	-	0.1±0.0	0.1±0.0
16:0	25.3±1.1	22.8±3.0	30.8±0.6	42.2±2.8	51.2±1.5	39.9 ±0.9
16:1n-7	2.3±0.2	3.1±0.9	2.9±0.1	1.9±0.0	1.9±0.3	4.1±0.4
i-17:0	-	-	-	-	-	0.1±0.1
ai-17:0	0.1±0.0	0.1±0.0	0.1±0.0	0.1±0.0	0.4±0.2	0.3±0.1
16:2	0.1±0.0	0.2±0.0	0.1±0.0	-	0.1±0.1	0.1±0.0
17:0	0.1±0.0	0.1±0.0	0.1±0.0	0.1±0.0	0.2±0.1	0.2±0.1
17:1	0.4±0.1	0.4±0.1	0.4±0.1	0.2±0.1	0.1±0.0	0.1±0.0
18:0	13.0±0.2	11.4±0.6	9.3±1.2	20.4±0.7	8.9±0.6	8.4±0.5
18:1n-9	2.9±0.4	3.5±1.7	5.8±5.7	3.3±0.8	6.0±1.6	6.8±1.5
18:1n-7	0.5±0.0	0.6±0.1	0.4±0.1	0.6±0.1	0.4±0.1	0.9±0.2
18:2	0.1±0.0	0.2±0.1	0.1±0.0	0.2±0.0	0.3±0.1	0.5±0.1
18:2n-6	0.9±0.1	1.0±0.4	1.2±0.5	0.6±0.1	1.4±0.4	1.6±0.4
19:0	0.4±0.1	0.4±0.0	0.3±0.1	0.3±0.1	0.2±0.1	0.2±0.0
18:3n-6	3.6±0.1	5.1±0.8	4.2±0.7	2.6±0.4	6.8±0.6	8.0±0.7
18:3n-3	0.1±0.0	-	-	-	-	0.1±0.0
18:4n-3	1.4±0.1	1.8±0.4	1.2±0.1	1.0±0.1	1.6±0.2	2.7±0.4
20:0	1.0±0.1	0.8±0.1	1.0±0.4	0.8±0.1	1.0±0.2	0.9±0.3
20:1n-9	2.2±0.0	1.8±0.1	1.5±0.4	1.1±0.0	1.7±0.1	2.3±0.3
20:1n-7	0.3±0.0	0.5±0.2	0.3±0.1	0.5±0.1	0.1±0.0	0.2±0.0
20:2n-6	0.6±0.1	0.4±0.0	0.4±0.1	0.3±0.1	0.1±0.1	0.4±0.2
20:3	-	-	0.7±0.1	0.4±0.1	0.4±0.1	-
20:3n-6	0.9±0.1	0.9±0.0	1.1±0.3	0.4±0.0	1.1±0.2	1.3±0.3
20:4n-6	6.7±0.4	14.7±2.1	10.4±0.8	1.8±0.2	2.0±0.3	2.3±0.3
20:4n-3	0.1±0.1	0.2±0.1	0.3±0.1	0.1±0.1	0.2±0.1	0.3±0.1
20:5n-3	16.5±0.4	9.5±1.3	10.3±0.7	9.9±2.1	1.7±0.3	3.0±0.5
22:0	0.3±0.1	0.2±0.0	-	0.2±0.0	0.1±0.0	-
22:1	0.2±0.1	0.1±0.0	0.2±0.1	0.2±0.1	0.2±0.1	0.2±0.1
22:2n-6	0.4±0.4	0.3±0.1	1.0±0.2	0.1±0.1	-	-
22:4n-6	5.5±0.6	7.2±1.2	4.1±1.1	2.6±0.6	1.1±0.2	1.3±0.3

22:5n-3	3.9±0.1	3.1±0.6	2.6±0.6	1.7±0.1	0.7±0.1	1.2±0.3
22:6n-3	6.3±0.4	6.2±0.1	4.9±1.0	3.3±0.4	4.1±0.3	4.2±0.4

Table S15. The total lipids FA composition (% of total, mean ± SD, *n* = 3) of coral of the families Pocilloporidae, Pectiniidae and Fungiidae collected at Vietnam coast in spring [103]. i = *iso*-, and ai = *anteiso*-FA

FA	Pocilloporidae			Pectiniidae	Fungiidae
	Stylophora pistillata	*Pocillopora damicornis*	*Seriatopora hystrix*	*Echinophyllia orpheensis*	*Sandalolitha robusta*
12:0	0.2±0.1	0.2±0.0	0.1±0.0	0.1±0.0	0.1±0.1
14:0	5.3±1.4	3.6±0.2	5.9±0.6	1.5±0.2	4.6±0.2
14:1	0.2±0.1	0.2±0.0	0.2±0.0	0.2±0.0	0.9±0.2
i-15:0	-	0.1±0.0	-	-	0.4±0.1
15:0	0.1±0.0	0.1±0.0	0.1±0.0	0.1±0.0	0.6±0.2
15:1	-	-	-	-	-
16:0	39.9±4.0	36.8±1.0	42.9±2.5	40.0±1.2	35.5±1.1
16:1n-7	3.8±0.5	2.2±0.1	3.9±0.0	2.2±0.2	4.8±0.5
i-17:0	-	-	-	-	0.6±0.1
ai-17:0	0.3±0.2	0.1±0.0	-	0.4±0.1	0.3±0.0
16:2	0.2±0.1	-	0.1±0.0	0.2±0.0	1.3±0.2
17:0	0.1±0.0	0.1±0.0	0.2±0.0	-	0.9±0.2
17:1	0.1±0.0	0.2±0.0	0.1±0.0	-	0.4±0.2
18:0	7.9±0.5	14.5±1.2	10.8±0.4	5.7±0.4	14.2±0.7
18:1n-9	7.2±1.7	4.8±0.5	5.3±0.1	20.3±0.8	8.0±0.5
18:1n-7	1.3±0.1	0.7±0.1	1.1±0.1	0.7±0.2	3.5±0.2
18:2	0.1±0.0	-	0.2±0.0	0.6±0.2	0.4±0.1
18:2n-6	0.6±0.1	1.3±0.1	0.5±0.0	1.0±0.3	1.4±0.2
19:0	-	0.1±0.0	-	-	0.5±0.1
18:3n-6	0.9±0.1	2.3±0.1	1.5±0.1	2.4±0.2	1.2±0.3
18:3n-3	0.2±0.1	0.2±0.0	0.1±0.0	-	0.1±0.0
18:4n-3	1.8±0.1	2.1±0.1	1.2±0.0	1.1±0.2	0.1±0.0
20:0	0.6±0.1	1.6±0.2	0.5±0.1	0.3±0.1	1.1±0.3
20:1n-9	1.0±0.5	1.5±0.1	0.4±0.1	1.7±0.2	1.4±0.2
20:1n-7	0.1±0.0	-	-	0.2±0.0	0.5±0.1
20:2n-6	0.4±0.1	0.4±0.0	0.2±0.0	0.5±0.1	0.7±0.2
20:3	0.1±0.0	0.3±0.2	-	-	0.2±0.1
20:3n-6	2.9±0.3	2.4±0.0	3.1±0.4	0.7±0.2	0.6±0.2
20:4n-6	5.1±0.2	3.9±0.0	3.5±0.5	3.4±0.3	4.2±0.3
20:4n-3	1.8±0.5	0.8±0.1	1.0±0.1	0.3±0.0	-
20:5n-3	1.8±0.1	3.0±0.3	1.8±0.2	1.7±0.2	1.6±0.1
22:0	-	0.3±0.1	-	-	0.2±0.1
22:1	-	-	-	0.3±0.2	0.5±0.2
22:2n-6	0.4±0.0	0.6±0.3	-	-	0.3±0.1

22:4n-6	1.9±0.1	2.6±0.1	1.2±0.1	1.7±0.2	1.4±0.2
22:5n-3	1.1±0.3	0.7±0.1	1.1±0.3	2.3±0.3	0.7±0.2
22:6n-3	13.2±2.1	12.3±0.1	13.3±2.2	9.2±0.4	2.6±0.2

Table S16. The total lipids FA composition (% of total, mean ± SD, *n* = 3) of corals of the families Poritidae and Faviidae collected at Vietnam coast in spring [103], and *Caulastrea tumida* (January, the South-China Sea, Vietnam) [92].

FA	Poritidae			Faviidae		
	Porites cylindrica	*Porites nigrescens*	*Porites lobata*	*Favia* sp. I	*Favia* sp. II	*Caulastrea tumida*
12:0	0.3±0.0	0.1±0.0	0.2±0.0	0.2±0.0	0.1±0.0	
14:0	2.3±0.2	1.5±0.0	2.3±0.2	4.9±0.4	5.0±0.4	2.5
i-15:0	-	-	0.2±0.0	-	0.2±0.0	
15:0	0.1±0.0	0.2±0.1	0.5±0.1	-	-	
16:0	36.3±10.0	40.8±2.8	35.4±3.4	40.4±2.4	38.7±2.9	41.1
16:1n-7	1.6±0.1	1.5±0.1	2.8±0.5	3.7±0.3	4.3±0.6	2.4
ai-17:0	0.1±0.0	0.2±0.1	0.2±0.0	0.3±0.1	0.2±0.1	0.1
16:2	0.1±0.0	0.1±0.0	0.2±0.1	0.1±0.0	0.3±0.0	0.2
17:0	0.1±0.0	0.1±0.0	0.3±0.0	-	-	
17:1	0.2±0.1	0.2±0.1	0.3±0.0	-	-	0.6
18:0	9.8±0.7	12.7±0.4	7.9±0.7	4.5±0.7	5.2±0.4	11.3
18:1n-9	15.1±1.3	9.4±0.1	19.0±1.2	7.6±0.4	9.1±0.9	3.4
18:1n-7	0.6±0.1	0.5±0.0	1.4±0.2	1.1±0.2	1.1±0.2	2.2
18:2	0.2±0.0	0.2±0.0	0.2±0.0	1.1±0.3	0.6±0.1	0.3
18:2n-6	1.1±0.1	0.6±0.1	1.5±0.2	1.6±0.2	3.0±0.2	0.8
18:3n-6	1.6±0.4	0.6±0.0	1.3±0.2	10.6±0.8	9.9 ±0.6	6.0
18:3n-3	0.1±0.0	-	0.1±0.0	-	-	
18:4n-3	1.7±0.7	1.6±0.1	0.6±0.1	1.2±0.2	1.1±0.1	0.8
20:0	0.5±0.1	0.5±0.0	0.4±0.1	0.4±0.0	0.6±0.1	1.6
20:1n-9	1.1±0.1	1.3±0.3	1.2±0.3	0.4±0.1	0.5±0.0	0.9
20:1n-7	0.5±0.4	0.4±0.1	-	-	-	0.3
20:2n-6	1.1±0.1	0.7±0.3	0.9±0.2	0.1±0.0	0.3±0.0	
20:3	0.1±0.0	0.6±0.6	-	0.1±0.0	-	
20:3n-6	0.4±0.1	0.2±0.0	0.6±0.1	1.9±0.3	2.1±0.2	1.9
20:4n-6	6.1±1.6	3.2±0.2	7.0±1.4	4.6±0.2	3.7±0.3	4.9
20:4n-3	0.2±0.0	0.2±0.0	0.2±0.0	0.2±0.0	0.2±0.1	0.2
20:5n-3	4.1±1.1	4.8±0.7	2.0±0.2	0.8±0.2	1.0±0.2	2.9
22:0	0.3±0.1	0.3±0.1	-	0.1±0.0	-	0.2
22:1	0.1±0.0	0.2±0.1	-	-	0.2±0.0	
22:2n-6	0.1±0.0	0.3±0.0	-	0.1±0.0	-	
22:4n-6	3.1±1.1	3.2±0.9	4.2±0.6	2.1±0.4	2.0±0.4	0.5
22:5n-3	1.3±0.4	1.5±0.4	2.1±0.5	6.7±0.5	6.2±0.6	1.9
22:6n-3	8.7±2.9	11.6±0.5	5.5±0.4	3.6±0.4	2.9±0.3	10.1
24:0	0.1±0.0	0.2±0.0	0.2±0.0	-	-	-

24:1n-9	0.1±0.0	0.1±0.0	0.1±0.0	-	-	-
24:2n-6	-	0.1±0.0	0.1±0.0	-	-	-
Δ5,9-24:2	0.1±0.0	0.1±0.0	0.2±0.0	-	-	-
Δ5,9,17-24:3	0.1±0.0	0.3±0.1	0.2±0.0	-	-	-
24:4n-3	0.1±0.0	0.2±0.1	0.2±0.1	-	-	-

Table S17. The composition of the main total lipids FA (%) of Vietnam hard corals [68].

FA	Coral species																			
	1	2	3	4	5	6	7	8	9	10	11	12	13	14	15	16	17	18	19	20
14:0	6.0	4.9	6.0	5.7	4.3	6.7	3.7	4.8	5.6	1.9	3.0	3.1	0.7	1.5	2.1	1.7	3.2	2.3	4.3	3.0
16:0	31.5	28.6	33.4	30.2	21.8	29.8	22.3	26.9	29.8	43.7	28.5	36.7	9.5	12.4	28.4	28.0	21.2	31.5	30.9	39.1
16:1n-7	3.7	2.8	3.4	3.0	2.1	3.9	1.8	3.4	3.5	1.7	3.1	2.8	3.2	3.3	1.9	2.7	3.2	2.2	2.5	4.2
7-Me-16:1n-10	0.1	0.1	0.1	0.1	0.1	0.1	0.1	-	0.1	-	0.1	-	1.8	2.3	0.6	0.6	0.5	0.3	0.2	-
18:0	3.8	6.4	4.4	5.8	7.4	4.7	6.1	3.5	3.0	5.7	6.2	6.6	6.1	8.2	8.2	7.1	5.7	7.0	6.4	4.8
18:1n-9	7.0	5.8	9.7	7.9	4.2	7.4	6.8	9.5	9.1	4.2	4.9	4.3	20.5	12.8	5.2	7.0	10.4	8.6	7.8	6.3
18:1n-7	0.3	0.3	0.4	0.4	0.3	0.5	0.2	0.5	0.4	1.2	1.1	1.0	3.4	2.7	0.7	1.0	1.9	1.3	1.4	1.0
18:2n-6	1.5	1.8	2.1	1.8	1.4	1.7	2.6	2.0	2.5	1.7	2.6	1.5	1.5	1.5	1.0	1.8	1.7	0.8	1.4	2.3
18:3n-6	10.9	9.7	10.8	9.7	8.0	9.4	12.5	12.4	15.0	9.3	11.9	10.5	0.9	0.8	7.1	10.2	5.6	4.6	5.3	6.2
18:4n-3	4.1	3.0	2.2	3.2	5.7	3.1	2.9	2.6	2.3	2.1	3.2	2.9	0.6	0.7	3.5	3.1	1.6	1.4	1.0	1.4
20:0	0.6	0.7	0.6	0.6	1.1	0.6	0.7	0.5	0.5	0.7	0.4	0.6	0.6	0.7	1.1	0.8	0.9	0.9	1.1	0.4
20:1n-9	3.3	2.9	3.4	3.8	3.3	3.2	3.8	4.0	3.5	0.3	0.2	0.1	1.9	1.7	3.2	3.9	6.3	4.6	3.8	0.2
20:1n-7	-	-	-	0.1	-	0.1	-	0.1	-	0.1	-	-	0.6	0.4	0.2	0.2	0.5	0.2	0.3	-
20:2n-6	0.4	1.0	0.5	0.8	0.7	0.5	0.7	0.5	0.5	0.4	0.2	0.2	0.9	0.8	0.6	0.5	0.4	1.1	0.3	1.0
20:3n-6	1.4	1.2	1.9	1.5	1.1	1.3	1.4	1.6	1.4	2.4	1.5	1.8	1.1	1.0	2.2	2.9	3.4	3.0	4.5	4.4
20:4n-6	4.1	6.8	2.8	4.2	9.9	3.2	7.3	2.6	2.5	5.6	10.0	6.8	10.9	11.7	11.1	9.8	7.9	4.8	6.3	10.1
20:4n-3	0.2	0.2	0.4	0.3	0.2	0.4	0.3	0.4	0.4	0.5	0.1	0.2	0.5	0.4	0.4	0.7	1.0	1.0	1.3	0.4
20:5n-3	3.7	3.1	2.9	3.5	6.2	5.6	5.7	4.1	4.0	1.2	2.6	1.3	7.3	7.1	3.1	2.1	1.8	1.8	1.3	2.4
22:0	0.1	0.7	-	0.1	0.3	0.1	0.2	0.1	0.1	0.1	0.2	0.4	-	-	0.3	0.1	0.1	-	0.1	0.2
22:1n-9	0.1	-	-	0.1	0.2	0.1	0.1	0.1	0.1	-	-	-	1.2	0.6	0.8	0.7	1.1	0.7	0.5	-
22:4n-6	1.8	3.4	1.8	2.5	5.2	2.0	3.8	1.7	1.3	3.1	3.9	2.7	6.7	8.4	4.9	3.6	2.9	1.5	1.8	1.8
22:5n-6	-	-	-	-	-	-	-	3.0	-	-	-	-	-	-	-	-	-	-	-	-
22:4n-3	-	-	-	-	-	-	-	0.6	-	-	-	-	-	-	0.1	0.2	0.2	-	0.3	-
22:5n-3	1.8	1.9	1.9	1.7	2.8	2.9	3.4	1.7	2.4	8.1	4.9	4.3	13.0	11.8	1.1	1.2	2.7	2.4	2.2	0.4
22:6n-3	7.6	7.0	7.1	5.9	6.4	6.4	9.1	9.3	8.7	3.6	7.9	6.4	1.7	1.2	5.9	6.2	10.0	12.9	9.7	9.6
Other	6.0	7.7	4.2	7.1	7.3	6.1	4.5	7.1	3.3	2.4	3.5	5.8	5.4	8.0	6.3	3.9	5.8	5.1	5.3	0.8

Table S17. (continued).

Fatty acid	Coral species																			
	21	22	23	24	25	26	27	28	29	30	31	32	33	34	35	36	37	38	39	40
14:0	2.4	5.0	2.6	5.4	3.3	2.0	3.6	2.9	4.4	3.0	3.2	4.7	2.7	1.7	2.7	3.2	2.9	3.2	2.8	2.2

16:0	37.5	32.6	27.5	55.2	32.8	34.7	25.6	37.1	25.8	35.7	21.2	31.8	18.4	34.1	60.6	51.0	40.9	49.3	27.2	31.3
16:1n-7	3.2	3.3	3.4	2.9	3.0	2.0	3.9	2.9	5.7	3.2	3.2	5.5	4.7	2.0	1.3	1.6	2.3	-	3.4	2.0
7-Me-16:1n-10	0.1	0.2	0.1	0.1	0.3	0.2	0.3	-	0.4	0.2	0.5	0.3	0.3	0.3	-	-	0.1	-	0.3	0.3
18:0	6.8	4.1	4.4	9.4	6.7	6.3	5.3	6.4	3.8	4.2	5.7	5.2	3.6	8.9	8.6	9.5	7.5	9.1	5.8	7.7
18:1n-9	5.7	5.1	6.8	3.9	7.9	6.6	6.2	4.8	6.3	7.5	10.4	7.9	8.8	5.3	3.4	3.3	4.9	4.3	8.6	8.3
18:1n-7	1.0	1.0	1.4	1.7	1.9	1.8	2.2	2.1	2.2	2.0	1.9	3.0	2.7	0.9	0.5	0.7	0.8	0.6	2.0	0.8
18:2n-6	2.1	3.8	2.7	1.3	2.3	1.4	2.1	1.2	2.2	1.9	1.7	2.2	2.8	1.5	1.1	1.0	2.2	1.2	3.6	2.2
18:3n-6	6.4	9.4	11.3	3.8	9.6	6.5	6.4	6.5	8.4	6.6	5.6	6.5	11.3	5.5	5.6	6.3	8.1	8.1	9.8	12.6
18:4n-3	1.8	2.2	2.6	0.4	1.3	2.4	3.3	2.8	2.3	2.1	1.6	1.7	2.7	1.5	0.6	0.8	1.3	0.5	1.7	1.5
20:0	0.6	2.3	0.7	0.5	0.5	0.7	0.6	0.8	0.5	0.5	0.9	0.4	0.5	1.1	0.9	0.9	0.8	1.0	0.6	1.1
20:1n-9	0.3	0.6	1.0	0.8	1.8	1.8	2.0	1.0	0.8	1.4	6.3	1.4	1.4	0.5	0.3	0.3	0.4	0.4	1.0	0.8
20:1n-7	-	0.1	0.2	0.2	0.2	0.2	0.3	0.2	0.3	0.2	0.5	0.3	0.2	-	-	-	-	-	0.2	-
20:2n-6	0.9	1.2	0.7	0.5	0.4	0.5	0.7	0.3	0.4	0.6	0.4	0.6	0.6	0.5	0.2	0.2	0.4	0.2	0.8	0.3
20:3n-6	3.3	1.9	3.3	1.3	2.6	3.1	3.0	2.3	2.1	2.5	3.4	1.2	3.2	1.8	1.7	2.2	3.0	3.2	3.6	3.1
20:4n-6	12.3	8.0	8.0	2.9	5.9	4.8	6.2	5.5	7.4	4.1	7.9	5.3	9.1	10.1	2.6	4.0	5.3	4.0	5.8	7.6
20:4n-3	0.3	0.2	0.5	0.1	0.3	0.5	0.6	0.5	0.3	0.4	1.0	0.2	0.6	0.1	0.1	0.2	0.2	0.1	0.5	0.2
20:5n-3	2.5	2.1	2.1	0.6	1.5	1.7	5.2	2.0	3.9	3.0	1.8	3.8	2.1	2.2	1.1	1.7	2.1	1.3	1.0	2.3
22:0	0.3	0.4	0.1	0.1	0.1	0.2	0.2	0.2	0.1	-	0.1	-	0.1	0.3	0.3	0.3	0.3	0.4	-	0.3
22:1n-9	-	0.3	0.2	0.1	0.5	0.3	0.3	0.1	0.1	0.3	1.1	-	0.3	0.1	-	-	-	0.1	0.2	-
22:4n-6	3.4	3.8	1.8	1.0	1.9	1.5	1.5	2.4	2.9	1.4	2.9	1.4	3.2	5.3	1.3	2.0	2.8	2.2	2.7	3.7
22:5n-6	-	-	-	-	-	-	-	-	-	-	-	-	-	-	-	-	-	-	-	-
22:4n-3	-	-	-	-	-	-	-	0.1	0.1	-	0.2	-	-	-	-	-	-	-	-	-
22:5n-3	0.4	2.6	1.1	0.5	2.2	0.5	0.3	0.9	3.0	0.7	2.7	3.5	7.4	4.3	1.5	2.1	2.5	1.9	9.4	1.3
22:6n-3	7.6	5.5	14.1	4.2	10.4	16.5	17.2	11.6	10.1	14.2	10.0	6.2	9.7	6.3	4.6	7.4	9.3	6.1	3.6	8.2
Other	1.1	4.3	3.4	3.1	2.6	3.8	3.0	5.4	6.5	4.3	5.8	6.9	3.6	5.7	1.0	1.3	1.9	2.8	5.4	2.2

Table S17. (continued).

Fatty acid	Coral species								
	41	42	43	44	45	46	47	48	49
14:0	2.1	3.4	3.6	2.8	5.4	2.1	1.5	1.0	3.6
16:0	28.7	29.2	41.0	16.0	37.0	25.7	17.3	26.2	24.2
16:1n-7	3.5	5.2	2.5	2.2	2.8	2.4	1.0	0.9	10.6
7-Me-16:1n-10	0.2	0.1	0.2	0.5	0.4	0.2	-	0.1	0.2
18:0	6.6	3.9	7.2	5.7	8.6	5.2	4.7	5.5	4.9
18:1n-9	6.5	12.3	4.3	5.4	4.5	16.9	21.5	21.0	4.4
18:1n-7	1.2	1.2	1.2	2.9	5.3	1.0	0.5	0.5	1.9
18:2n-6	1.5	3.2	3.0	1.9	1.1	1.1	1.2	0.7	3.0
18:3n-6	13.5	9.7	6.8	5.6	3.7	3.3	2.3	2.2	11.0
18:4n-3	1.0	2.0	1.8	2.0	0.6	2.0	3.8	2.2	2.2
20:0	0.8	0.4	0.6	0.9	0.6	0.3	0.3	0.2	0.6
20:1n-9	0.5	1.0	0.4	4.3	2.5	4.0	4.6	1.6	0.1
20:1n-7	-	0.1	0.1	1.0	1.2	0.1	-	-	0.2
20:2n-6	0.4	0.6	0.3	1.4	1.0	0.8	1.4	0.9	0.6
20:3n-6	4.0	3.2	2.1	5.8	2.7	0.8	0.4	0.9	3.6
20:4n-6	7.1	6.8	5.9	11.2	6.6	6.1	7.8	7.4	9.4
20:4n-3	0.2	0.4	0.3	1.6	0.3	0.2	0.2	0.4	0.2
20:5n-3	2.4	1.7	1.2	2.9	0.9	4.4	2.9	2.6	2.0
22:0	0.2	-	0.1	0.3	0.1	0.1	-	0.2	0.1
22:1n-9	0.1	0.1	0.1	1.3	0.6	0.5	0.5	0.3	-
22:4n-6	3.5	1.0	2.8	2.2	1.6	2.9	5.8	4.8	5.5
22:5n-6	-	-	-	-	-	-	-	-	-
22:4n-3	-	-	-	0.1	-	-	-	-	1.4
22:5n-3	2.1	1.5	9.4	0.5	0.2	1.4	6.1	2.6	0.1
22:6n-3	10.9	8.3	2.7	14.8	8.3	14.7	11.3	14.7	6.2
Other[b]	3.0	4.7	2.4	6.6	4.0	3.8	4.9	3.1	4.0

Species numbers as is showed in Table 10. b 12:0, 14:1, i-15:0, ai-15:0, 15:0, 15:1, i-16:0, ai-16:0, 16:1п-9, 16:1n-5, i-17:0, 16:2n-7, 17:0, 16:3n-4, i-18:0, 18:2, 18:2n-7, 19:0, 18:3n-4, 20:2n-7, 22:2NMI, 22:3n-6, 22:1n-7.

Table S18. The FA composition (%) of total lipids of three gorgonian corals species of Vietnam [87].

Fatty acid	*Psammogorgia nodosa*	*Bebryce indica*	*Mopsella aurantia*	Fatty acid	*Psammogorgia nodosa*	*Bebryce indica*	*Mopsella aurantia*
14:0	1.7	2.2	1.3	20:1	1.1	-	-
15:0	-	-	1.0	20:4n-6	5.1	29.1	40.9
16:0	52.7	15.1	6.4	20:4n-3	-	1.1	-
16:1	2.4	4.0	3.1	20:5n-3	-	4.1	7.6
16:2	1.5	4.4	-	22:1	1.1	-	-
17:0	-	2.1	3.1	22:3n-9	-	4.5	-
18:0	16.5	10.9	5.8	22:3n-6	-	-	1.1

18:1	4.8	7.6	5.4	22:4n-6	-	-	1.4
18:3n-6	-	-	2.5	22:5n-6	-	2.2	4.9
18:4n-3	-	1.1	1.1	22:5n-3	-	-	1.4
20:0	11.0	1.6	1.0	22:6n-3	-	5.0	3.8

Table S19. The total lipids FA composition (%) of the gorgonian *Paragorgia arborea*, alcyonarians *Eunephthya* sp. and *Sarcophyton* sp. [104].

Fatty acid	Gorgonaria	Alcyonaria	
	Paragorgia arborea	*Eunephthya* sp.	*Sarcophyton* sp.
14:0	1.1	1.9	1.3
15:0	-	0.2	0.3
15:1	-	0.1	-
i-16:0	-	0.1	0.2
16:0	7.1	7.2	29.6
16:1	3.2	2.3	2.4
i-17:0	-	0.4	-
ai-17:0	-	0.6	4.6
17:0	0.6	1.2	6.6
17:1	0.5	0.2	1.1
i-18:0	0.5	0.2	-
18:0	3.4	0.6	7.8
18:1n-9	11.1	21.1	1.7
18:1n-7	-	0.9	-
18:2n-6	0.3	0.9	1.4
18:3n-3	-	0.9	0.3
18:4n-3	0.4	1.4	4.7
20:0	-	1.7	-
20:1	9.6	8.2	-
20:2n-6	0.2	0.6	0.3
20:3n-3	-	0.7	0.3
20:3n-6	-	0.6	2.0
20:4n-3	0.7	2.8	2.1
20:4n-6	20.2	5.4	15.7
20:5n-3	8.7	14.1	1.6
22:1n-9	6.1	1.6	-
22:2n-6	-	0.5	-
22:4n-6	0.8	0.2	4.9
22:5n-6	0.4	0.1	0.6
22:5n-3	0.4	0.9	-
22:6n-3	1.9	9.9	1.7
24:1n-9	0.8	1.7	-

24:5n-6	15.6	2.6	5.3
24:6n-3	6.0	7.6	1.5

Table S20. The total lipids FA composition (%) of gorgonians (winter) from the Nha Trang Bay (Vietnam, the South China Sea) [46].

Fatty acid	*Euplexaura erecta*	*Nicaule crucifera*	Plexauridae spp. 1	Plexauridae spp. 2	*Subergorgia suberosa*	*Junceella fragilis*	*Junceella juncea*	*Rumphella aggregata*
14:0	1.8	0.9	2.3	1.0	1.4	0.3	1.2	1.5
15:0	0.4	0.7	-	-	1.3	-	-	-
16:0	9.0	7.2	9.5	6.9	10.4	18.9	7.0	13.5
16:1	3.4	2.9	3.5	2.3	4.4	0.8	2.6	2.5
16:2	4.5	1.9	5.0	2.2	5.2	1.1	2.5	6.3
16:3	-	-	0.3	-	5.1	-	0.2	-
18:0	5.1	5.2	6.3	3.5	7.3	12.2	6.9	4.5
18:1	3.5	4.7	3.7	5.3	11.9	3.5	3.2	2.3
18:2n-6	0.7	0.8	0.8	1.6	1.1	4.4	1.0	1.2
18:3n-6	-	1.2	0.4	-	-	-	0.4	1.9
18:3n-3	0.8	2.1	0.9	0.6	0.9	0.3	1.1	0.9
20:0	1.0	0.2	1.0	0.9	1.6	7.9	1.1	6.0
20:3n-6	0.2	-	-	0.4	0.7	-	-	1.7
20:4n-6	41.3	33.4	43.3	50.5	27.3	25.7	47.9	40.5
20:4n-3	0.6	-	0.3	2.5	0.8	-	-	0.7
20:5n-3	7.8	4.7	4.3	6.6	3.1	6.2	4.6	4.0
22:4n-6	0.7	1.0	1.1	1.9	1.4	8.7	8.4	1.6
22:5n-3	-	1.2	-	0.6	1.2	0.3	0.8	-
22:6n-3	3.1	5.1	2.2	1.4	3.0	7.4	2.0	2.7
24:5n-6	1.2	2.7	1.1	1.8	2.0	1.5	2.0	0.5
24:5n-3	12.0	18.4	13.2	9.6	7.6	-	6.7	7.1
24:6n-3	2.3	1.9	-	-	-	-	-	-

Table S21. The total lipids FA composition (%) of alcyonarians from the Nha Trang Bay (Vietnam, the South China Sea) [46,106].

Fatty acid	*Sinularia flexibilis*	*Sinularia capillosa*	*Sarcophyton crassocaule*	*Sinularia leptoclados*	*Sinularia* sp.	*Sarcophyton* aff. *glaucum*	*Sarcophyton* sp. 1	*Sarcophyton* sp. 2	*Sarcophyton* sp. 3	*Cladiella* sp. 1	*Cladiella* sp. 2
14:0	0.8	0.9	1.3	0.5	-	2.2	8.0	2.5	3.9	6.0	1.6
15:0	-	1.1	-	-	-	-	0.9	0.6	0.4	0.4	0.1
16:0	29.1	20.5	28.3	53.5	27.2	30.5	50.9	57.6	30.2	26.6	44.7

16:1	1.0	2.0	1.4	0.3	0.9	2.1	5.8	4.2	6.5	6.6	3.6
16:2	6.1	6.2	3.8	7.1	6.7	8.9	0.9	0.9	0.6	0.4	0.1
17:0	-	-	-	-	-	-	0.7	7.5	0.5	1.5	8.6
18:0	6.2	5.0	8.2	7.0	6.1	7.8	8.1	10.4	5.4	4.9	14.0
18:1	1.3	6.3	1.5	1.8	3.3	2.6	5.0	3.1	3.5	4.1	4.1
18:2n-6	4.2	2.6	2.1	3.7	4.3	3.8	3.0	2.2	4.4	1.4	0.3
18:3n-6	-	-	-	-	-	3.4	5.5	6.1	12	14.5	-
18:3n-3	0.8	0.7	0.7	2.0	0.5	1.7	0.2	0.2	0.4	0.1	0.2
18:4n-3	-	-	0.8	-	-	-	0.8	1.3	3.2	0.7	1.7
20:0	6.9	6.8	6.1	4.4	4.8	7.6	0.7	1.2	0.6	0.9	1.2
20:1	0.8	26.3	0.6	1.2	13.0	0.6	0.3	0.1	0.1	0.3	0.1
20:3n-6	-	-	-	-	-	-	0.2	0.1	0.5	4.0	0.3
20:3n-3	-	-	-	-	-	-	0.1	0.2	-	0.4	0.3
20:4n-6	30.1	14.9	30.4	11.2	18.2	17.6	3.5	3.6	11.0	8.3	3.3
20:4n-3	1.9	-	0.3	1.0	0.7	0.4	0.2	0.4	1.0	1.6	1.1
20:5n-3	3.3	1.1	1.9	1.1	1.4	2.6	1.1	0.9	4.0	3.3	1.2
22:4n-6	-	0.7	-	-	-	-	-	0.4	0.1	0.3	-
22:5n-3	-	-	-	-	-	2.2	0	0.3	0.2	0.3	-
22:6n-3	2.1	1.6	2.3	2.1	0.6	1.0	3.7	0.8	6.1	5.1	1.3
24:5n-6	-	-	-	-	-	-	4.2	1.0	2.0	1.6	0.5
24:5n-3	5.0	2.6	9.3	2.6	10.6	4.2	-	-	-	-	-
24:6n-3	-	-	-	-	-	-	1.0	0.5	1.7	1.6	-

Table S22. The FA composition (%, mean ± SD) of the total (TL, *n* = 6), neutral (NL, *n* = 4) and polar (PL, *n* = 4) lipids of the cold-water alcyonarian *Gersemia rubiformis* from the Bering Sea.

Fatty acid	TL	NL	PL
14:0	1.0 ± 0.4	1.4 ± 0.1	0.6 ± 0.1
15:0	0.3 ± 0.1	0.3 ± 0.1	0.2 ± 0.0
15:1	0.2 ± 0.1	0.1 ± 0.0	0.6 ± 0.5
16:0	7.4 ± 2.2	10.4 ± 1.9	5.5 ± 2.1
16:1n-9	0.5 ± 0.2	0.6 ± 0.0	0.3 ± 0.0
16:1n-7	2.9 ± 0.8	3.8 ± 0.0	1.2 ± 0.3
16:1n-5	0.1 ± 0.0	0.1 ± 0.0	0.1 ± 0.0
i-17:0	0.2 ± 0.1	0.2 ± 0.1	0.2 ± 0.0
16:2	0.3 ± 0.2	0.3 ± 0.1	0.1 ± 0.0
br-17:1	0.7 ± 0.1	1.3 ± 0.3	0.4 ± 0.2
16:3	0.5 ± 0.1	1.0 ± 0.3	0.4 ± 0.0
17:0	0.1 ± 0.1	0.2 ± 0.1	0.1 ± 0.0
17:1n-9	0.9 ± 0.8	0.2 ± 0.2	0.4 ± 0.2
18:0	1.4 ± 0.5	1.4 ± 0.4	1.3 ± 0.3
18:1n-9	3.8 ± 1.0	5.8 ± 1.3	1.5 ± 0.2

18:1n-7	3.0 ± 0.9	3.5 ± 0.2	2.0 ± 0.1
18:2n-6	1.0 ± 0.3	2.0 ± 0.7	0.4 ± 0.1
br-19:1	0.2 ± 0.1	0.2 ± 0.0	0.1 ± 0.0
18:3n-3	0.3 ± 0.1	0.7 ± 0.3	0.1 ± 0.0
19:2+18:4n-3	0.8 ± 0.2	1.4 ± 0.4	0.2 ± 0.1
20:1n-11	0.8 ± 0.2	1.1 ± 0.1	0.4 ± 0.1
20:1n-9	0.9 ± 0.1	1.4 ± 0.6	0.4 ± 0.1
20:1n-7	9.5 ± 1.4	5.7 ± 0.4	7.2 ± 2.7
Δ5,11-20:2	0.2 ± 0.1	0.3 ± 0.1	0.1 ± 0.0
20:2n-6	0.6 ± 0.1	0.8 ± 0.2	0.4 ± 0.1
20:3n-9	0.6 ± 0.2	0.6 ± 0.1	0.2 ± 0.0
20:4n-6	22.8 ± 4.7	10.8 ± 0.3	39.1 ± 3.2
20:3n-3	0.4 ± 0.2	0.5 ± 0.1	0.2 ± 0.0
20:4n-3	1.5 ± 0.7	2.2 ± 0.3	0.3 ± 0.1
20:5n-3	16.5 ± 3.4	23.1 ± 6.4	13.6 ± 3.9
22:1n-11	0.6 ± 0.1	0.7 ± 0.2	0.3 ± 0.1
22:1	0.2 ± 0.1	0.4 ± 0.1	-
22:2n-3	0.6 ± 0.2	0.8 ± 0.0	0.3 ± 0.1
22:4n-6	0.9 ± 0.4	0.4 ± 0.0	1.2 ± 0.6
22:5n-6	0.2 ± 0.1	0.3 ± 0.0	0.4 ± 0.2
22:4n-3	0.3 ± 0.2	0.4 ± 0.1	-
22:5n-3	1.6 ± 1.1	1.7 ± 0.2	0.5 ± 0.3
22:6n-3	2.0 ± 0.3	3.0 ± 0.0	2.4 ± 0.7
24:5n-6	5.9 ± 1.3	2.2 ± 0.2	11.4 ± 2.1
24:6n-3	4.4 ± 1.2	4.3 ± 0.5	4.3 ± 1.8

Table S23. The main FA composition (%) of total lipids the genus *Dendronephthya* [68,79].

Fatty acid	*Dendronephthya*											
	crystallina[1]	*crystallina*[1]	*aurea*[1]	*aurea*[1]	*gigantean*[1]	*involuta*[1]	*cervicornis*[2]	*pulchella*[2]	sp. 1[1]	sp. 2[1]	sp. 3[1]	sp. 4[1]	sp. 5[2]
14:0	2.5	2.7	1.6	2.1	1.4	1.5	1.3	1.7	1.3	1.0	1.9	1.1	1.0
16:2n-7	0.3	-	1.1	-	-	-	1.1	1.6	0.7	-	-	-	0.6
16:1n-9	1.9	1.0	0.3	0.4	0.3	1.8	0.6	0.3	0.6	0.6	0.7	1.1	0.4
16:1n-7	3.1	2.5	2.5	1.8	2.5	1.6	1.3	1.5	2.0	1.3	1.9	1.2	0.9
16:0	16.3	18.0	12.8	13.4	13.5	14.0	11.2	12.7	7.5	6.7	11.8	7.3	10.0
7-Me-16:1	4.4	7.0	3.4	7.3	7.0	3.6	5.9	5.0	2.9	3.6	4.6	4.5	3.8
br-17:0	2.2	1.9	0.7	2.0	1.2	3.3	0.8	0.9	1.6	3.1	1.9	2.8	1.0
18:4n-3	1.1	0.3	1.1	0.1	0.3	-	0.6	0.8	1.1	0.6	0.6	0.6	0.4
18:2n-7	-	-	0.7	-	-	2.8	0.5	-	-	3.6	1.2	3.6	
18:2n-6	1.4	1.3	0.9	1.0	1.3	-	1.4	1.1	1.0	1.8	1.8	1.9	0.8
18:1n-9	4.5	5.1	2.7	3.0	5.0	5.2	3.9	3.3	2.4	3.1	3.9	3.4	3.0
18:1n-7	3.3	4.1	2.2	2.5	4.3	2.8	1.8	1.6	1.2	2.5	3.6	3.6	1.1

18:0	8.8	1.5	6.2	9.3	9.1	6.4	7.7	6.4	3.4	4.3	6.2	4.9	5.7
20:4n-6	15.7	27.0	29.4	28.9	21.1	25.0	23.4	28.3	37.7	30.9	23.4	21.3	34.7
20:5n-3	3.3	3.5	3.3	1.9	1.5	1.8	2.5	3.7	3.8	1.3	1.6	1.6	3.4
20:3n-6	0.2	0.3	0.3	0.2	0.6	-	0.6	-	0.5	0.9	0.6	0.7	0.4
20:4n-3	0.3	0.2	0.6	0.1	0.7	-	0.3	0.5	0.5	1.1	0.7	1.6	0.6
20:1	0.3	1.3	0.6	0.7	2.3	-	0.4	-	-	0.8	1.1	1.2	-
20:0	0.8	0.1	0.3	0.1	0.2	0.7	1.0	0.4	-	0.6	1.0	0.4	0.5
22:5n-6	1.2	0.6	1.9	0.4	0.9	1.1	1.8	1.1	1.8	2.3	3.2	1.9	1.2
22:6n-3	4.1	2.3	3.9	0.9	1.3	2.1	3.7	2.5	4.1	2.2	3.0	2.5	2.5
22:4n-6	0.4	0.3	-	0.3	0.9	-	0.8	1.1	0.8	-	-	-	1.5
22:5n-3	-	-	0.5	-	0.3	1.3	-	-	-	0.9	0.7	0.7	-
24:5n-6	9.6	9.4	11.6	12.9	12.4	12.5	16.5	11.8	15.0	16.5	12.3	15.2	13.0
24:6n-3	3.6	2.2	4.3	2.3	4.7	2.4	2.8	2.4	4.6	5.1	4.0	7.1	4.1
15:0,17:0, 19:0	2.9	3.1	1.6	2.7	2.4	2.9	2.4	2.0	1.0	1.7	2.7	2.0	2.8

Table S24. The main FA composition (%) of total lipids of the genus *Sinularia* [110].

Fatty acid	*S. leptoclados*	*S. flexibilis*	*S.* aff. *deformis*	*S. lochmodes*	*S.* cf. *muralis*	*S. densa*	*S. notanda*	*S. cruciata*
14:0	1.2	1.8	1.4	2.3	1.8	2.1	1.7	1.4
16:0	26.1	27.2	20.6	21.5	22.7	37.4	26.6	20.7
7-Me-16:1	1.3	1.3	0.3	3.6	0.6	1.1	1.3	0.2
16:1n-9	0.6	tr	0.3	0.6	0.5	0.3	0.2	0.3
16:1n-7	1.1	2.7	2.0	2.2	2.6	3.5	2.2	1.7
16:2n-7	3.5	8.6	6.0	10.9	11.2	7.3	6.8	6.5
16:3n-4	1.2	1.3	0.9	1.2	0.6	0.5	0.8	1.0
16:4n-1	1.0	0.9	0.7	0.9	0.7	0.5	0.6	0.8
18:0	10.8	6.6	12.9	2.4	7.6	6.3	8.1	7.1
18:1n-9	1.7	1.6	2.2	1.8	3.1	2.6	1.6	1.7
18:1n-7	0.2	tr	0.9	0.0	0.3	0.5	0.4	0.8
18:2n-7	3.3	5.3	6.9	8.4	4.1	2.6	5.4	3.9
18:2n-6	tr	0.2	0.2	0.0	0.0	0.6	0.1	0.2
18:3n-6	0.7	1.4	0.4	0.6	0.9	0.8	0.5	0.8
18:4n-3	1.1	7.2	5.6	4.4	4.5	4.8	5.2	6.2
20:0	1.0	0.1	0.8	0.0	1.2	1.1	0.4	0.6
20:3n-6	1.1	0.8	0.4	1.5	0.9	0.7	0.9	1.3
20:4n-6	23.2	16.9	19.1	21.2	18.1	10.2	19.1	23.8
20:3n-3	0.9	0.2	0.6	0.7	0.5	0.3	0.3	0.3
20:4n-3	0.8	1.1	0.9	1.3	1.0	0.6	1.0	1.1
20:5n-3	1.0	2.1	2.4	2.3	1.5	0.8	2.2	2.2
22:6n-3	2.5	3.0	2.9	2.7	3.4	1.9	3.1	3.9
24:5n-6	8.9	5.3	5.8	6.0	6.3	5.6	7.3	8.4

24:6n-3	0.9	1.1	1.8	0.9	1.2	1.2	1.2	1.2

Table S25. The main FA composition (% of the sum) of total lipids of the genus *Sinularia* [79].

Fatty acid	*S.* cf. *robusta*	*S.* aff. *exilis*	*S. brassica*	*S. brassica*	*S. erecta*	*S.* aff. *polydactila*	*S. siaesensis*	*S. siaesensis*	*S. gibberosa*	*Sinularia sp.*	*S. polydactila*	*S. flexibilis*
14:0	2.7	3.9	5.8	5.7	2.0	1.6	3.5	4.7	1.4	2.6	1.4	1.4
16:0	23.1	25.4	19.0	24.1	23.0	23.8	17.3	22.2	29.8	46.7	30.1	44.7
16:1n-7	2.8	4.6	3.8	4.2	1.9	1.4	4.1	3.9	2.8	-	1.7	3.5
16:2n-7	9.4	0.9	0.4	0.3	6.0	9.1	2.2	1.5	9.3	5.6	5.7	6.5
7-Me-16:1	0.4	-	0.3	0.3	0.8	0.5	-	0.1	0.2	-	0.2	0.5
16:4	0.8	0.8	1.7	1.2	1.8	2.3	1.4	0.9	0.6	0.2	0.4	0.3
18:0	3.9	6.4	3.3	3.5	6.5	4.8	5.8	5.3	10.3	6.7	11.0	7.1
18:1n-9	1.9	2.4	2.8	4.0	1.4	0.7	2.1	2.8	3.6	1.5	2.9	2.5
18:1n-7	0.2	0.9	0.3	0.4	0.1	-	0.6	0.4	0.9	0.1	0.5	0.3
18:2n-7	1.0	0.6	-	0.1	2.3	1.4	1.3	0.6	6.5	1.3	5.0	3.2
18:2n-6	0.4	1.5	1.8	1.8	0.8	-	0.9	1.0	-	0.4	0.6	-
18:3n-6	2.9	14.1	8.4	9.2	2.4	-	10.0	11.7	-	-	0.5	-
18:4n-3	4.1	2.8	3.3	2.1	2.3	3.2	2.6	3.0	2.7	2.1	2.6	2.7
20:0	0.6	0.6	0.5	0.9	1.0	0.7	0.5	0.4	0.7	2.7	1.2	0.6
20:1n-9	0.2	0.1	0.2	-	0.1	-	0.1	0.1	-	0.2	0.2	-
20:3n-6	0.6	0.9	1.1	1.3	1.0	0.5	0.8	0.7	0.7	0.7	1.1	0.4
20:4n-6	16.8	12.7	17.4	13.0	22.7	23.6	22.5	15.4	11.8	10.8	13.6	9.0
20:4n-3	0.7	1.1	0.4	0.5	1.2	0.7	0.8	0.6	1.6	0.9	1.1	1.2
20:5n-3	3.4	3.3	3.9	3.1	3.2	3.6	3.5	6.2	0.8	0.6	1.0	0.8
22:0	0.4	0.5	0.3	0.4	0.5	0.4	0.3	0.4	0.4	2.2	1.3	0.4
22:4n-6	0.4	0.3	-	-	0.4	0.6	0.6	0.3	0.4	0.3	0.5	-
22:6n-3	6.4	7.9	8.2	11.8	3.0	3.7	4.7	5.5	1.6	1.3	1.7	2.0
24:5n-6	5.1	4.1	5.9	4.2	5.6	7.4	6.8	4.1	4.2	3.7	5.2	3.5
24:6n-3	1.8	2.0	3.0	2.8	2.0	2.2	2.5	3.1	1.1	0.7	1.0	1.1

Table S26. The main FA composition (%) of total lipids of the genus *Sarcophyton* [68].

Fatty acid	*S. buitendijki*	*S. cinereum*	*S.* aff. *crassum*	*S. buitendijki*	*S. trocheliophorum*	*S. acutum*	*S. elegans*
14:0	2.6	3.1	3.1	2.1	1.8	2.6	2.0
16:2n-7	7.8	12.6	11.8	8.3	9.1	6.5	6.4
16:1n-9	0.7	0.4	0.6	1.3	1.0	-	0.4
16:1n-7	1.8	2.8	2.6	1.3	1.8	2.9	1.8
16:0	37.8	37.9	40.4	19.2	28.7	19.5	31.6
7-Me-16:1n-10	1.9	0.7	1.4	1.4	2.2	1.4	0.6
18:3n-6	-	-	-	-	-	1.9	-
18:4n-3	4.7	4.8	2.4	8.1	6.7	8.9	3.7
18:2n-7	1.3	1.5	1.5	0.9	1.6	1.0	1.5

18:2n-6	-	0.1	0.3	0.3	0.3	0.5	1.3
18:1n-9	1.3	1.4	1.9	2.1	1.8	1.7	2.4
18:1n-7	0.2	0.2	0.2	-	0.3	0.3	-
18:0	7.5	5.2	5.6	4.9	7.1	5.7	4.6
19:1	-	0.2	0.2	-	-	-	3.1
20:4n-6	16.3	12.6	15.1	24.8	17.9	21.1	15.2
20:5n-3	2.2	1.5	1.0	5.2	2.2	5.0	6.2
20:3n-6	0.2	0.2	0.2	0.2	0.6	0.3	1.1
20:4n-3	0.3	0.3	0.3	0.1	0.4	0.2	2.8
20:1	0.2	0.3	0.2	1.2	0.2	0.3	-
20:0	0.8	0.7	0.6	-	0.4	0.5	1.3
22:5n-6	0.1	0.1	0.1	-	0.1	0.2	-
22:6n-3	3.1	2.5	1.3	5.5	3.8	5.1	1.5
22:5n-3	0.2	-	-	-	-	0.3	-
22:4n-3	0.1	-	0.3	-	0.1	-	0.6
22:0	0.3	0.3	0.3	-	0.1	0.4	0.4
24:5n-6	4.4	4.2	4.6	8.4	5.6	8.8	4.8
24:6n-3	0.9	0.6	0.5	0.8	0.8	0.5	0.4

Table S27. The main FA composition (%) of total lipids of the genus *Sarcophyton* [68].

Fatty acid	*S. crassum*	*S. ehrenbergi*	*S.* cf. *glaucum*	*S. regulare*	*S. cinereum*	*S. spongiosum*	*Sarcophyton* sp.
14:0	1.5	1.8	2.5	1.7	3.4	2.1	1.9
16:0	30.2	41.6	21.3	35.4	22.9	25.2	28.4
16:1n-7	2.7	2.4	3.9	2.6	4.5	2.2	1.8
16:2n-7	6.3	9.3	16.4	5.9	14.0	8.0	6.1
7-Me-16:1n-10	0.8	1.4	0.8	1.3	1.7	1.5	2.1
16:4	0.1	0.5	0.9	0.1	-	0.2	0.2
18:0	8.0	4.4	1.0	5.3	6.0	5.2	7.1
18:1n-9	3.4	2.5	1.4	2.8	8.5	1.6	1.9
18:1n-7	0.4	0.1	-	0.2	2.4	0.1	0.2
18:2	0.9	1.0	0.8	0.5	1.0	0.8	0.4
18:2n-7	2.4	1.0	1.1	0.7	1.0	1.6	1.3
18:2n-6	0.2	-	-	0.2	0.3	0.2	0.1
18:3n-6	-	0.4	0.2	0.7	0.5	0.2	0.2
18:4n-3	2.8	3.5	6.4	4.5	4.4	4.5	4.0
20:0	1.1	0.4	0.2	0.7	0.3	0.5	0.6
20:1	-	-	-	0.2	1.9	-	-
20:3n-6	0.7	-	0.2	0.3	-	0.2	0.2
20:4n-6	19.6	11.5	19.6	18.0	11.1	25.2	22.9
20:4n-3	0.8	0.5	0.6	0.5	0.2	0.3	0.4

20:5n-3	0.9	1.0	1.7	1.2	1.4	2.4	2.1
22:0	0.5	0.2	-	0.3	-	0.2	0.2
22:4n-6	0.2	0.1	-	0.4	0.2	0.2	0.3
22:5n-6	0.1	0.4	0.4	0.2	0.5	0.1	0.2
22:6n-3	1.8	1.7	4.1	2.2	2.1	3.0	2.6
24:5n-6	6.9	3.2	5.5	7.0	2.1	6.8	7.9
24:6n-3	0.8	1.2	0.6	0.7	0.6	1.0	0.9

Table S28. The main FA composition (%) of total lipids of the genus *Lobophytum*. [68,79].

FA	*L. pusillum*[1]	*L.* cf. *delectum*[1]	*L. michaelae*[2]	*L. ransoni*[2]	*L. batarum*[2]	*L. crassum*[2]	*Lobophytum* sp. [2]
14:0	2.0	1.1	2.3	1.2	4.3	1.8	-
16:0	29.9	16.8	30.0	24.8	25.0	36.8	29.6
16:1n-7	2.9	0.9	2.0	1.4	4.9	2.1	2.8
16:2n-7	7.0	3.9	4.9	4.2	0.6	6.5	13.4
7-Me-16:1n-10	2.0	1.9	1.8	1.3	4.9	0.9	2.3
18:0	6.2	6.1	6.3	5.9	7.6	6.7	3.8
18:1n-9	2.5	2.0	1.6	1.8	3.8	2.7	2.4
18:1n-7	0.4	-	0.5	0.2	2.4	0.2	0.2
18:2n-7	2.3	2.4	3.7	1.4	-	0.9	2.0
18:2n-6	0.5	1.6	-	0.2	1.2	0.1	0.3
18:3n-6	-	0.4	0.1	-	0.1	-	0.3
18:4n-3	4.1	0.6	1.9	3.7	0.4	3.7	4.6
20:0	1.4	0.7	0.5	0.7	0.7	1.2	0.2
20:1n-9	0.7	0.7	0.1	0.2	0.2	0.2	0.1
20:3n-6	0.8	0.7	0.1	0.2	0.2	0.3	0.2
20:4n-6	18.4	30.4	22.2	24.1	21.6	13.5	16.1
20:4n-3	0.5	-	0.6	0.7	0.2	0.5	0.9
20:5n-3	1.3	0.2	1.0	3.4	2.2	1.3	1.9
22:0	0.6	0.4	0.3	-	0.1	0.4	0.1
22:4n-6	-	-	0.4	-	0.4	0.1	-
22:5n-6	0.2	0.1	-	-	0.4	-	0.2
22:5n-3	-	0.4	-	-	0.1	-	-
22:6n-3	2.5	0.2	1.5	2.9	1.3	2.2	2.6
24:5n-6	7.2	14.9	5.5	7.6	2.3	4.0	4.7
24:6n-3	0.9	0.4	1.4	2.1	0.4	0.8	1.2

Table S29. The main FA composition (%) of the total lipids of some alcyonarian species with zooxanthellae [68,79].

Fatty acid	*Cladiella laciniosa*	*Cladiella laciniosa*	*Cladiella subtilis*	*Cladiella pachyclados*	*Lytophyton* sp.	*Cespitularia* sp.	*Clavularia* sp.

14:0	4.9	7.4	7.3	5.7	1.0	6.2	2.3
16:0	21.0	34.6	27.8	17.5	13.6	22.6	14.7
16:1n-7	4.3	4.0	3.4	4.1	0.5	7.4	1.6
16:2n-7	0.5	0.3	0.3	0.3	0.1	1.9	-
7-Me-16:1n-10	0.3	0.2	0.3	0.2	0.5	0.2	0.3
18:0	4.0	8.3	7.8	12.0	6.9	3.1	2.7
18:1n-9	5.4	4.9	4.7	2.6	30.7	2.0	4.0
18:1n-7	0.4	0.4	0.2	0.2	0.2	0.1	0.3
18:2n-6	2.3	2.6	1.4	1.5	0.1	1.9	6.3
18:3n-6	14.2	9.4	0.1	0.2	-	11.5	0.5
18:4n-3	7.4	3.7	4.5	8.3	5.7	7.4	8.3
20:0	0.4	0.1	0.2	0.1	0.5	-	0.4
20:1	0.4	0.2	0.2	0.2	1.0	0.1	-
20:3n-6	0.3	0.3	0.2	0.3	-	0.2	-
20:4n-6	12.9	9.4	10.6	18.4	15.0	15.8	21.6
20:4n-3	0.2	0.1	0.2	0.2	0.1	0.1	-
20:5n-3	3.6	2.1	3.6	4.3	6.7	4.4	7.5
22:5n-6	0.9	0.1	-	0.1	0.1	-	-
22:4n-3	-	-	-	-	-	0.1	2.0
22:5n-3	-	-	-	0.1	-	-	2.6
22:6n-3	6.0	5.2	5.3	5.9	7.2	6.1	10.5
24:5n-6	4.3	2.2	3.5	3.4	5.0	5.4	2.4
24:6n-3	1.3	0.9	2.1	0.5	0.6	0.5	0.4
24:5n-3	-	-	-	-	-	-	2.4
24:4n-3	-	-	-	-	0.1	0.3	2.3

Table S30. The main FA composition (%) of total lipids of *Heliopora coerulea* and other alcyonarians [68].

Fatty acid	*Heliopora coerulea*	*Carijoa riisei*	*Carijoa riisei*	*Klyxum molle*	*Lemnalia* cf. *exilis*	*Lemnalia* cf. *peristyla*	*Nephthea capnelliformis*	*Nephthea* sp.	*Nephthea* sp.
14:0	4.9	1.9	2.7	4.3	1.5	2.4	2.2	2.1	1.3
16:0	45.5	7.4	20.4	13.6	20.3	18.1	16.2	11.1	13.6
16:1n-7	2.3	0.7	0.6	5.2	0.9	0.6	0.4	0.5	0.3
16:2n-7	0.2	0.3	-	0.4	-	-	0.2	1.2	-
7-Me-16:1n-10	0.1	0.3	0.3	0.4	0.2	0.3	0.8	10.8	1.9
16:4	-	0.2	0.2	0.4	0.2	0.2	0.3	0.5	0.2
18:0	6.6	4.7	24.6	6.0	15.8	15.5	7.7	9.3	9.1
18:1n-9	2.4	4.9	8.2	5.0	4.1	4.2	33.3	26.8	32.1
18:1n-7	0.8	-	0.2	0.3	0.1	0.1	0.2	0.3	0.3
18:2n-6	3.2	1.8	1.6	1.7	2.4	1.3	-	2.6	-
18:3n-6	1.0	9.4	4.8	10.6	7.9	4.4	-	0.3	-
18:4n-3	2.7	10.6	4.6	8.7	2.6	5.2	1.0	1.0	0.6
20:0	0.5	0.2	2.7	0.1	3.2	1.4	0.7	1.2	1.0

20:1n-9	0.6	0.2	0.3	0.2	0.2	0.2	0.8	1.0	1.4
20:3n-6	0.3	0.9	0.9	0.3	1.3	0.7	-	-	0.1
20:4n-6	0.6	17.7	7.4	19.7	18.0	2.4	12.5	11.4	15.2
20:4n-3	0.2	1.3	0.7	0.3	0.7	1.1	-	0.1	0.2
20:5n-3	10.2	11.4	5.8	4.8	5.9	27.2	4.4	1.5	3.9
22:0	0.1	-	1.0	-	0.8	0.5	-	0.2	0.2
22:4n-6	-	0.7	0.2	0.1	0.4	0.4	0.3	-	0.1
22:5n-6	0.9	-	-	0.1	0.2	-	-	-	0.2
22:5n-3	1.8	-	0.1	-	-	0.3	-	-	0.2
22:6n-3	9.9	7.3	3.2	6.9	3.2	4.6	9.1	5.2	7.4
24:5n-6	-	8.9	4.0	5.5	4.8	3.1	4.8	4.1	6.9
24:6n-3	2.0	2.1	0.8	1.2	1.6	2.4	1.0	0.6	1.4

Table S31. The main FA composition (%) of total lipids of gorgonians [111].

FA	*Acabaria erithraea*	*Acanthogorgia isoxia*			*Chironephthya variabilis*			*Echinog-orgia* sp.	*Ellisella plexauroides*			*Menella praelonga*			*Paralemnalia thyrsoides*[a]		*Rumphella aggregata*[a]	
14:0	0.8	1.7	1.6	1.4	2.0	1.8	1.5	2.9	1.5	1.0	1.5	1.2	0.6	1.3	4.5	3.5	2.0	1.2
i-15:0	-	-	-	-	-	0.1	-	-	-	-	-	-	-	-	-	-	-	-
ai-15:0	-	0.1	0.1	0.1	0.1	0.1	0.2	-	-	0.1	0.1	-	-	0.1	-	-	-	-
15:0	0.4	0.7	0.8	0.9	0.8	0.5	0.9	0.9	0.6	0.4	0.5	0.5	0.2	0.8	0.2	0.5	0.3	0.1
16:2n-7	-	-	-	-	-	-	-	-	-	-	-	-	-	-	-	-	2.8	1.6
ai-16:0	0.2	0.2	0.2	0.2	0.1	0.3	0.2	-	0.2	0.2	0.2	0.2	-	0.2	-	-	-	-
16:1n-9	0.5	0.4	0.5	0.1	0.2	0.3	1.6	1.0	2.1	0.2	0.4	0.3	-	0.1	-	1.5	0.2	0.2
16:1n-7	1.1	1.3	1.9	3.1	2.0	1.5	1.0	1.6	0.4	1.1	1.7	1.5	1.1	3.0	1.0	0.7	3.0	2.3
16:1n-5	0.5	0.2	-	0.8	0.3	0.3	0.2	-	-	0.3	0.3	0.3	-	0.6	0.1	-	-	0.2
16:0	7.5	10.9	10.1	14.6	10.6	12.2	10.4	14.0	9.9	7.6	9.2	9.0	2.4	15.1	27.8	19.6	33.4	32.5
7-Me-16:1	1.4	1.6	2.6	1.7	6.7	1.8	1.7	2.9	3.3	2.2	2.1	2.9	1.7	1.7	0.2	0.2	2.3	3.1
i-17:0	0.1	0.3	0.6	0.7	0.7	0.5	0.5	0.5	1.0	0.7	0.5	0.8	-	0.8	0.2	-	-	-
2-Me-16:0	0.1	0.2	-	0.1	-	0.1	-	-	-	-	-	-	-	0.2	-	0.1	0.5	0.3
ai-17:0	0.2	0.2	0.1	0.2	-	0.2	0.2	-	0.3	0.2	0.2	0.3	-	0.3	-	-	-	-
17:0	0.6	0.9	0.7	1.8	0.7	0.8	1.0	0.9	1.2	1.5	1.3	1.1	-	2.0	0.3	0.3	0.1	0.1
18:3n-6	-	0.1	0.1	0.1	-	-	-	-	-	-		-	-	0.1	8.7	8.7	1.4	0.8
18:4n-3	0.4	0.1	0.1	0.3	-	0.1	-	-	0.1	0.2	0.1	0.1	0.5	0.2	1.8	3.9	3.0	1.3
br-18:0	0.3	0.1	0.1	0.1	-	0.2	0.2	-	0.2	0.2	0.3	0.3	-	0.2	-	-	-	-
18:2n-9	-	-	-	-	-	-	0.2	-	-	-	-	-	-	-	0.3	0.3	0.5	0.5
18:2n-7	-	-	-	-	-	-	-	-	-	-	-	-	-	-	-	-	2.6	1.9
18:2n-6	0.9	0.9	0.8	1.4	0.7	2.4	1.0	0.7	0.6	0.9	1.2	0.8	0.3	1.4	1.7	1.4	0.8	0.5
18:1n-9	2.4	3.9	2.2	3.7	2.4	6.7	3.0	3.1	2.4	2.5	3.1	2.0	0.5	3.9	3.6	3.1	3.5	4.2
18:1n-7	1.9	1.1	1.5	3.8	1.8	1.8	1.3	2.2	1.8	2.5	3.3	1.2	0.5	4.1	0.2	0.1	0.4	0.3
18:0	6.5	6.3	4.5	7.7	5.3	5.7	6.4	5.3	5.1	8.8	8.3	6.1	0.9	8.9	12.4	8.6	8.2	11.0
ai-19:0	0.1	-	-	-	-	0.1	-	-	-	0.1	-	0.1	-	0.1	-	-	-	-

Me-F18	2.7	-	-	-	0.5	-	-	-	0.5	0.1	-	0.3	1.0	-	-	-	-	-
19:0	0.3	0.3	0.3	0.5	-	0.3	0.3	-	0.2	0.5	0.4	0.4	-	0.6	0.1	0.1	-	0.1
20:4n-6	37.2	43.7	48.2	24.4	34.9	40.2	46.2	47.6	42.3	37.3	38.3	40.6	57.0	21.5	22.4	28.7	13.8	12.5
20:5n-3	1.7	4.9	3.5	1.4	2.5	2.0	1.2	2.2	2.6	1.3	2.0	2.7	7.4	0.9	1.9	3.6	2.6	1.5
20:3n-6	0.4	0.4	-	1.4	-	0.3	0.3	0.2	0.2	0.5	0.3	-	-	1.4	0.4	0.5	1.6	1.1
20:4n-3	0.3	0.2	0.1	0.5	-	0.2	-	0.2	-	0.3	0.5	0.1	0.3	0.4	-	0.1	0.7	0.5
20:2n-6	0.4	-	-	0.5	-	0.1	-	-	-	0.2	0.2	-	-	0.6	-	-	-	0.1
20:1n-9	0.4	0.4	1.0	1.3	0.6	0.3	0.6	-	-	0.6	0.5	0.3	-	1.6	0.2	-	0.4	0.4
20:1n-7	0.2	-	0.5	0.5	-	0.2	0.3	-	-	0.4	0.7	-	-	0.6	-	-	-	0.1
diMe-F18-3	-	-	-	-	0.7	-	-	-	0.8	-	-	0.4	-	-	-	-	-	-
20:0	0.3	0.3	0.6	0.7	0.3	0.3	0.4	-	0.3	0.6	0.3	0.4	-	0.8	0.9	0.7	5.3	8.1
Me-F20	-	-	-	-	0.9	0.3	0.3	-	0.4	1.0	-	0.5	0.6	-	-	-	-	-
21:0	-	-	0.1	0.3	-	0.1	-	-	0.1	0.3	-	0.2	0.1	0.4	0.1	0.1	0.2	0.3
22:5n-6	5.7	0.4	0.2	2.8	0.5	0.8	0.8	0.2	0.3	1.2	1.2	0.4	0.4	3.0	-	-	0.1	0.1
22:6n-3	3.9	1.6	0.8	5.2	1.5	1.4	1.2	0.8	1.6	3.2	3.9	1.3	1.8	4.7	2.3	2.6	1.8	1.4
22:4n-6	0.6	0.5	0.4	10.6	0.4	0.3	1.4	0.4	0.4	14.7	11.8	0.5	0.7	10.6	0.1	0.2	0.7	0.6
22:5n-3	0.3	0.2	0.1	-	-	0.1	0.1	-	0.1	0.5	0.7	0.1	-	0.6	-	-	-	-
22:3n-6	-	-	-	0.5	-	-	-	-	-	-	-	-	-	0.5	-	-	0.1	0.1
diMe-F20	-	-	-	-	0.8	0.2	0.2	-	0.5	0.6	0.2	0.5	0.6	-	-	-	-	-
diMe-F20-3	-	-	-	-	0.8	-	-	-	1.1	-	-	0.3	-	-	-	-	-	-
22:2n-6	-	0.2	-	-	-	-	-	-	-	-	-	-	0.3	0.2	-	-	0.3	0.5
22:1n-9	0.2	0.1	-	0.4	-	0.1	-	-	-	0.4	0.5	0.2	-	0.6	0.1	-	0.3	0.5
22:1n-7	-	-	-	0.2	-	-	0.3	-	-	0.2	-	-	-	0.3	-	-	0.1	0.1
22:0	-	-	1.6	0.3	-	-	-	-	-	0.2	0.2	0.1	-	0.4	0.3	0.3	3.3	4.4
Me-F22	0.1	-	-	-	1.7	0.2	0.2	-	1.6	0.9	0.3	2.0	0.5	-	-	-	-	-
24:5n-6	14.5	11.2	10.7	0.6	12.4	12.6	12.0	8.9	8.9	0.2	0.2	13.3	13.5	0.6	6.5	8.3	3.2	3.6
24:6n-3	2.3	3.0	2.4	1.8	1.6	1.2	1.1	2.6	1.3	1.6	1.3	2.4	4.8	1.6	0.6	1.0	0.2	0.2
diMe-F22	0.3	-	-	-	5.1	0.2	0.3	-	4.9	0.5	0.1	3.7	1.2	-	-	-	-	-
24:1n-9	-	-	-	0.2	-	-	-	-	-	0.3	-	-	-	0.3	-	-	0.1	0.1
24:1n-7	0.6	-	-	-	-	-	-	-	-	-	-	-	-	0.1	-	-	-	-
24:0	-	0.3	-	0.2	-	0.1	-	-	-	-	-	-	-	0.2	-	-	0.1	0.2

Me-F18 10,13-epoxy-11-methyloctadeca-10,12-dienoic acid, *Me-F20* 12,15-epoxy-13-methyleicosa-12,14-dienoic acid, *diMe-F20* 12,15-epoxy-13,14-dimethyleicosa-12,14-dienoic acid, *Me-F22* 14,17-epoxy-15-methyldocosa-14,16-dienoic acid, *diMe-F22* 14,17-epoxy-15,16-dimethyldocosa-14,16-dienoic acid, *diMe-F18-3* 12,15-epoxy-13,14-dimethyloctadeca-12,14-dienoic acid, *diMe-F20-3* 14,17-epoxy-15,16-dimethyleicosa-14,16-dienoic acid. [a] Species with zooxanthellae.

Table S32. The main FA composition (%) of total lipids of the gorgonian *Bebryce studeri* [111].

Fatty acid	Content	Fatty acid	Content	Fatty acid	Content
14:0	1.7±0.7	18:0	5.4±1.1	24:5n-6	7.2±2.5
i-15:0	1.8±0.2	ai-19:0	0.2±0.0	24:6n-3	0.5±0.2
ai-15:0	0.2±0.1	19:0	0.5±0.1	Furan acids	
15:0	0.5±0.3	20:4n-6	21.7±7.2	Me-F18	0.2±0.1
ai-16:0	0.4±0.2	20:5n-3	2.0±0.9	Me-F20	0.5±0.3
16:1n-9	0.3±0.1	20:3n-6	0.3±0.1	diMe-F20	0.2±0.1

16:1n-7	1.9±0.8	20:4n-3	0.1±0.1	Me-F22	0.9±0.6
16:1n-5	0.3±0.1	20:1n-9	0.4±0.1	diMe-F22	0.9±0.9
16:0	8.9±2.7	20:1n-7	0.3±0.1	Sponge markers	
7-Me-16:1n-10	2.3±0.6	20:0	2.3±1.4	24:1n-9	0.3±0.1
i-17:0	1.3±0.7	21:0	0.5±0.2	24:1n-7	0.6±0.4
ai-17:0	0.6±0.2	22:5n-6	4.2±2.3	24:0	0.6±0.4
17:0	0.8±0.2	22:6n-3	3.5±1.8	25:2(5,9)	0.8±0.1
18:4n-3	0.3±0.3	22:4n-6	1.1±0.5	26:3(5,9,19)	0.8±0.5
br-18:0	0.2±0.1	22:5n-3	0.5±0.3	26:2(5,9)	12.9±2.8
18:2n-6	0.7±0.1	22:1n-9	0.3±0.1	26:2	0.7±0.4
18:1n-9	1.8±0.5	22:1n-7	0.2±0.0	26:1	1.3±0.4
18:1n-7	2.1±0.4	22:0	0.9±0.5	28:3(5,9,19)	2.7±0.9

Table S33. The main FA composition (%) of total lipids of gorgonians.* species with zooxanthellae [68].

Fatty acid	*Siphonogorgia variabilis*	*Siphonogorgia* cf. *harrisoni*	*Siphonogorgia* cf. *harrisoni*	*Annella mollis*	*Annella mollis*	*Mopsella* sp.	*Mopsella* cf. *spinosa*	*Parisis* cf. *minor*	*Menella* cf. *praelonga*	*Menella* cf. *praelonga*	*Menella flora*
14:0	1.9	2.2	2.2	2.2	1.9	1.6	1.6	1.4	1.6	1.2	2.2
16:0	13.1	21.3	14.5	13.2	11.8	11.0	9.8	13.2	9.8	8.3	12.3
16:1n-7	2.1	3.0	2.1	1.8	1.7	1.3	1.7	1.7	1.3	1.9	2.2
16:2n-7	0.3	-	-	0.3	0.4	0.8	0.6	0.5	0.6	1.0	0.4
7-Me-16:1	4.3	9.5	6.1	5.5	5.6	0.9	1.2	2.4	1.6	2.0	1.9
16:4	-	-	-	0.2	0.2	3.6	0.8	2.1	1.5	1.7	1.8
18:0	6.3	10.0	8.5	6.8	5.6	7.8	6.6	9.0	3.9	3.9	4.8
18:1n-9	3.4	5.3	4.2	2.6	2.5	2.9	2.8	3.6	3.1	3.3	2.9
18:1n-7	2.2	1.6	2.3	2.1	2.1	1.0	1.4	2.2	1.2	2.5	2.0
18:2n-7	-	1.6	1.4	-	-	-	0.3	-	-	-	-
18:2n-6	0.9	-	-	1.0	0.9	0.7	1.0	0.8	1.1	1.2	0.9
18:3n-6	1.0	0.6	0.2	0.3	0.3	0.2	0.7	0.4	0.3	0.5	0.4
18:4n-3	-	0.8	0.4	0.5	0.8	0.6	1.1	0.7	0.2	0.5	0.5
20:0	0.9	1.2	0.9	0.5	0.5	0.2	0.3	1.1	0.6	0.7	0.6
20:1n-9	1.9	0.4	0.4	0.2	0.2	0.5	0.2	0.5	0.3	0.3	0.3
20:3n-6	0.8	0.3	0.5	1.0	1.0	0.6	0.6	0.7	0.3	0.8	0.5
20:4n-6	34.8	21.8	27.1	19.2	19.3	32.4	28.0	22.3	41.2	34.3	36.7
20:4n-3	-	-	0.4	1.5	1.3	0.3	0.6	0.4	-	0.7	-
20:5n-3	2.1	1.6	1.5	2.1	2.8	1.6	3.8	4.9	2.7	2.4	4.6
22:0	-	0.4	0.4	0.4	0.3	-	0.1	0.3	-	1.3	-
22:4n-6	-	0.6	0.5	2.9	2.5	0.7	0.9	1.0	0.8	1.2	1.1
22:5n-6	0.8	0.4	0.9	3.2	3.0	1.5	1.5	4.4	0.4	0.6	-
22:4n-3	-	-	-	0.2	0.2	-	0.3	-	-	-	-
22:5n-3	0.9	0.7	0.2	0.2	0.3	-	0.5	-	0.7	1.1	0.7

22:6n-3	1.6	1.4	1.7	7.1	7.7	1.9	4.1	5.1	1.5	2.2	3.0
24:5n-6	11.9	6.4	13.1	10.6	11.3	15.4	13.0	5.8	14.3	13.1	11.3
24:6n-3	1.7	0.6	3.4	2.8	4.4	2.6	6.8	5.5	2.1	4.4	2.8

Table S33. (continued).

Fatty acid	*Echinogorgia* cf. *gracillima*	*Paracis* cf. *horrida*	*Hicksonella princeps**	*Hicksonella princeps**	*Viminella* cf. *petila*	*Viminella* cf. *crassa*	*Narella* sp.	Plexauridae spp. 1	Plexauridae spp. 2
14:0	1.9	1.1	2.5	1.9	1.3	1.6	1.9	1.3	1.4
16:0	10.7	7.8	29.8	35.8	6.1	8.1	14.6	9.6	9.5
16:1n-7	1.9	2.1	3.1	2.5	2.1	2.1	1.9	1.9	1.3
16:2n-7	0.4	-	0.3	0.1	0.2	0.2	-	0.3	0.8
7-Me-16:1n-10	2.1	3.6	1.7	1.6	2.2	2.0	1.2	2.5	1.8
16:4	1.6	-	0.3	0.2	1.0	0.6	-	0.3	0.6
18:0	5.3	4.4	4.6	5.8	5.5	5.8	5.9	5.3	5.7
18:1n-9	2.4	5.3	3.5	4.2	3.5	3.7	6.4	3.8	3.4
18:1n-7	1.5	3.2	0.2	0.1	3.1	2.7	1.6	1.6	1.4
18:2n-6	0.8	-	3.0	2.9	1.6	1.5	2.1	0.4	0.5
18:3n-3	0.5	-	0.3	0.2	1.4	1.2	0.4	0.1	0.2
18:4n-3	0.7	0.2	3.3	1.6	1.8	1.4	0.2	0.2	0.2
20:0	0.4	0.4	4.1	7.1	1.4	0.5	0.4	0.8	0.5
20:1n-9	0.2	0.7	0.4	0.4	1.1	0.7	1.9	1.0	0.5
20:3n-6	0.4	0.5	0.3	0.5	1.5	0.8	-	0.3	0.2
20:4n-6	37.6	34.2	17.8	12.5	16.5	25.6	11.6	24.3	34.3
20:4n-3	0.3	0.4	0.1	-	2.1	1.2	-	0.3	0.1
20:5n-3	3.6	2.3	5.2	3.2	2.5	3.0	15.9	6.8	3.5
22:0	-	0.2	2.3	4.9	0.6	0.2	-	0.3	0.2
22:4n-6	0.8	0.9	1.3	1.1	9.1	9.2	0.7	2.9	0.9
22:5n-6	0.5	2.3	0.4	0.7	5.0	3.1	1.9	2.2	2.1
22:4n-3	-	-	-	-	1.9	0.5	-	-	-
22:5n-3	0.5	0.7	0.3	-	1.9	1.4	0.9	1.5	0.6
22:6n-3	3.6	5.3	5.5	6.1	5.8	6.8	8.6	10.7	5.5
24:5n-6	12.0	13.4	2.0	2.4	1.7	0.5	-	8.8	13.5
24:6n-3	2.8	3.4	0.2	0.3	7.6	4.6	12.4	1.1	1.4

Table S34. The FA composition (%) of polar and neutral lipids of the hard coral *Stylopora pistillata* (Seychelles) at different depths [100].

Fatty acid	Polar lipids				Neutral lipids			
	Depth, m							
	3	9	35	25	3	9	35	25
14:0	0.8	1.2	0.8	0.7	3.6	2.8	0.8	1.0
16:0	4.2	4.2	4.3	5.8	36.9	24.1	23.7	21.5

16:1n-7	1.0	1.5	0.3	1.7	3.4	4.3	1.6	1.0
16:2	-	0.4	0.4	0.5	0.2	0.8	0.5	0.3
18:0	5.6	11.1	8.6	9.8	9.7	7.1	5.6	5.3
18:1n-9	0.8	1.5	2.4	2.3	4.3	11.2	17.7	16.5
18:2n-6	0.5	0.7	1.0	1.1	1.1	1.1	2.8	3.4
18:3n-6	0.9	1.1	8.1	7.7	2.4	1.2	0.8	0.8
18:4n-3	4.9	8.6	10.9	14.5	0.7	0.5	0.4	0.4
20:0	0.3	0.4	1.0	1.3	0.8	0.9	0.4	0.4
20:1	-	0.4	0.4	0.5	0.5	0.8	1.8	1.6
20:2n-6	3.7	1.9	0.9	1.3	0.5	0.6	1.2	1.4
20:3n-6	1.6	0.5	-	1.8	7.9	8.2	5.2	4.7
20:4n-6	25.3	19.0	25.0	17.0	2.0	1.8	1.1	1.4
20:4n-3	0.3	-	-	-	0.7	0.7	0.6	0.6
20:5n-3	23.8	19.9	11.8	12.0	3.3	0.7	0.5	0.5
22:3n-6	-	-	-	-	0.6	0.9	0.6	0.8
22:4n-6	9.4	10.5	8.4	8.4	0.6	0.7	1.0	1.1
22:5n-6	-	-	-	-	-	-	-	-
22:5n-3	2.6	3.6	1.6	1.9	1.9	1.7	1.8	2.0
22:6n-3	9.0	6.6	8.4	7.5	18.2	22.9	31.1	35.1

Table S35. The FA composition (%) of polar lipids of cnidarians, Okinawa Is., Japan [36].

Fatty acid	*Pocillopora damicornis*	*Pocillopora verrucosa*	*Stylophora pistillata*	*Montipora aequituberculata*	*Acropora microphthalma*	*Porites lutea*	*Porites cylindrica*	*Fungia fungites*	*Galaxea fascicularis*	*Goniastrea aspera*	*Oulastrea crispata*	*Tubastrea* sp.	*Lobophytum crassum*
14:0	3.7	3.6	5.4	1.6	1.3	0.9	1.0	1.4	2.7	1.5	2.8	0.5	1.8
16:0	48.2	49.1	45.2	38.7	36.5	19.2	15.8	43.5	54.8	51.9	32.2	19.5	30.9
16:1n-7	3.0	1.7	3.3	2.7	2.3	1.2	0.4	1.4	3.9	1.3	1.6	0.5	2.6
2-OH-16:0	0.9	1.1	0.7	1.5	0.8	1.1	0.6	1.4	0.5	0.0	1.0	0.6	0.6
18:0	9.3	9.1	6.7	6.1	13.4	5.5	4.4	14.6	7.5	11.6	20.3	16.4	12.3
18:1n-9	6.6	3.8	6.2	4.2	2.8	5.6	4.3	5.7	5.7	4.5	5.9	2.8	2.1
18:2n-6	1.9	0.9	0.4	1.2	1.3	1.2	3.4	1.1	1.2	0.8	1.0	1.6	6.1
18:3n-6	2.4	0.7	1.5	8.2	8.3	14.5	6.2	7.7	4.7	2.0	2.1	0.3	15.9
18:4n-3	3.7	0.9	10.2	5.8	3.1	8.2	15.2	1.1	1.1	0.9	7.0	0.2	0.0
20:0	3.9	2.3	0.9	0.6	1.4	0.5	0.5	2.8	2.1	1.5	1.8	1.2	0.8
20:1	0.0	0.0	1.6	0.3	2.9	0.7	0.4	1.4	0.3	0.5	1.6	1.0	0.0
20:2	0.0	0.0	0.3	0.3	0.5	0.8	0.7	0.1	0.4	0.3	0.5	1.0	0.0
20:3n-6	1.6	1.0	1.2	0.4	1.1	1.0	0.3	1.3	1.6	0.3	1.2	1.7	1.0

20:4n-6	0.0	0.0	0.5	3.4	7.2	15.6	14.1	4.5	2.1	2.5	4.8	14.0	20.4
20:5n-3	0.0	0.0	4.7	1.3	8.9	5.6	4.3	0.8	0.2	0.0	2.8	9.5	4.2
22:0	1.0	1.1	0.7	0.3	0.3	0.5	0.2	0.9	1.1	0.0	0.1	0.0	0.7
22:4n-3	0.8	1.2	0.7	0.0	0.0	6.6	6.9	2.8	0.7	0.2	1.7	6.4	1.1
22:5n-3	0.6	1.2	1.0	3.4	1.8	1.9	1.2	0.3	0.7	0.3	1.0	14.4	0.4
22:6n-3	1.2	0.6	2.4	0.9	1.5	1.4	0.9	0.6	0.7	0.0	0.5	0.4	1.8

Table S36. The FA composition (%) of polar lipids of the gorgonian genus *Gorgonia, Pseudopterogorgia,* and *Eunicea* (Puerto-Rico) [45].

Fatty acid	*Gorgonia* (Gorgoniidae)	*Pseudopterogorgia* (Gorgoniidae)	*Eunicea* (Plexauridae)
14:1n-5	1.2 ± 0.1	0.6 ± 0.1	0.9 ± 0.4
14:0	4.6 ± 0.1	4.1 ± 1.0	2.7 ± 0.5
16:1n-7	0.5 ± 0.1	0.6 ± 0.3	6.3 ± 0.7
16:0	14.0 ± 4.3	17.4 ± 1.8	9.0 ± 2.0
17:0	0.1 ± 0.1	-	0.1 ± 0.1
18:3n-6	13.2 ± 2.6	7.3 ± 2.7	14.2 ± 3.3
18:4n-3	13.6 ± 2.8	12.0 ± 2.0	14.3 ± 5.4
18:2n-6	9.4 ± 0.9	4.6 ± 1.7	2.0 ± 1.2
18:1n-9	4.5 ± 0.5	3.0 ± 1.5	3.9 ± 2.4
18:0	4.3 ± 0.9	6.7 ± 0.7	3.4 ± 1.2
20:4n-6	9.7 ± 0.3	17.2 ± 2.4	13.1 ± 1.2
20:5n-3	3.8 ± 2.6	0.2 ± 0.2	1.7 ± 0.5
20:0	0.5 ± 0.1	0.9 ± 0.2	0.8 ± 0.5
22:6n-3	7.1 ± 1.5	6.1 ± 0.7	5.1 ± 1.0
22:4n-6	1.2 ± 0.9	-	0.7 ± 0.6
22:0	1.8 ± 1.8	0.6 ± 0.0	0.7 ± 0.6
24:5n-6	2.7 ± 1.3	10.2 ± 3.7	5.2 ± 3.6
24:6n-3	1.2 ± 0.8	3.7 ± 1.0	1.6 ± 1.0
n-6/n-3	1.4 ± 0.0	1.8 ± 0.3	1.6 ± 0.6

Table S37. The main FA composition (%) of the total lipids of Vietnamise hydrocorals [68].

Fatty acid	*Millepora dichotoma*	*Millepora platyphylla*
14:0	2.3	3.1
16:0	19.8	23.6
16:1n-7	-	0.1

7-Me-16:1n-10	0.4	0.5
18:0	15.3	15.4
18:1n-9	3.9	6.1
18:1n-7	-	0.3
18:2n-6	0.1	0.5
18:3n-6	-	0.2
18:4n-3	1.9	1.5
20:0	5.5	3.3
20:1n-9	0.4	0.2
20:2n-6	0.1	0.3
20:3n-6	0.3	0.3
20:4n-6	-	0.7
20:5n-3	0.8	0.4
22:0	0.5	0.2
22:1n-9	0.3	0.2
22:4n-6	3.5	2.6
22:5n-6	7.3	6.8
22:5n-3	1.1	0.4
22:6n-3	33.3	32.0

Table S38. The main FA composition (%) of the zooxanthellae total lipids of hermatypic corals [112].

Fatty acid	*Acropora divaricata*	*Acropora formosa*	*Acropora byercinthus*	*Acropora millepora*	*Acropora nasuta*	*Leptastrea pruinosa*	*Montastrea curta*	*Pocillopora damicornis*
14:0	4.3	3.3	5.4	3.5	3.7	3.4	8.6	4.8
16:0	18.8	19.0	18.2	23.1	16.6	18.1	21.6	20.0
16:1	2.0	3.9	3.4	2.0	2.9	3.9	2.9	2.8
18:0	4.4	4.4	6.4	6.9	4.7	7.1	5.2	7.2
18:1	2.1	3.3	3.2	3.4	1.9	3.7	4.1	2.8
18:2n-6	1.0	0.5	1.4	0.9	0.8	0.9	1.0	0.3
18:3n-6	14.5	9.2	7.4	6.2	8.1	8.3	8.3	2.8
18:3n-3	0.2	0.4	1.0	0.9	0.4	0.1	0.1	0.9
18:4n-3	10.4	15.8	9.2	10.1	12.7	9.2	9.3	18.1
18:5n-3	6.7	4.3	4.8	4.6	7.6	5.3	5.9	1.8
20:4n-6	2.4	2.7	4.6	2.8	2.3	7.8	5.7	6.3
20:5n-3	20.9	17.4	17.3	20.0	22.8	16.1	15.2	9.0
22:4n-6	1.2	1.1	2.7	1.9	1.3	2.6	1.5	3.9
22:5n-3	0.4	0.2	1.2	1.8	0.8	0.2	0.1	0.1
22:6n-3	7.5	7.9	7.1	7.9	8.4	6.4	7.1	16.0

Table S39. The main FA composition (%) of zooxanthellae galactolipids from hermatypic corals MGDG, and DGDG [112].

Fatty acid	*Acropora divaricata*		*Acropora formosa*		*Acropora byercinthus*		*Acropora millepora*		*Acropora nasuta*		*Leptastrea pruinosa*		*Pocillopora damicornis*	
	MGDG	DGDG	MGDG	DGDG	MGDG	DGDG	MGDG	DGDG	MGDG	DGDG	MGDG	DGDG	MGDG	DGDG
14:0	1.0	10.8	2.7	1.7	2.4	4.9	2.1	2.0	0.3	1.2	4.4	12.6	1.6	2.4
16:0	2.7	2.2	2.3	1.1	3.3	2.3	3.6	2.8	2.4	1.7	1.3	2.0	2.1	3.9
16:1	1.2	0.8	2.2	0.9	1.5	1.2	2.1	1.2	1.4	0.9	2.6	1.4	1.1	1.3
18:0	0.5	0.5	0.3	0.3	0.3	0.3	2.6	1.3	0.5	0.4	0.1	0.4	0.2	0.4
18:1	0.8	0.8	1.4	1.3	1.1	1.2	2.5	1.8	1.0	1.2	1.4	1.2	2.1	1.3
18:2n-6	1.0	0.6	0.5	0.3	0.7	0.6	2.0	0.4	0.7	0.5	0.7	0.5	0.6	0.1
18:3n-6	26.0	17.0	12.7	10.3	15.1	11.3	7.1	8.6	14.9	12.4	13.0	10.4	1.1	1.5
18:3n-3	-	0.1	-	0.1	-	0.7	2.0	0.1	0.1	0.2	-	-	0.1	0.1
18:4n-3	23.4	18.6	39.3	31.9	32.0	22.7	27.5	24.7	29.6	24.3	25.8	21.9	66.8	55.7
18:5n-3	20.8	10.9	14.9	8.8	23.9	13.4	18.2	12.2	24.0	13.5	22.7	10.9	10.8	6.1
20:4n-6	0.1	0.3	-	0.1	0.1	0.1	2.0	0.6	-	-	0.1	0.1	-	0.1
20:5n-3	21.2	35.0	19.2	34.7	18.9	36.6	19.9	36.1	22.9	40.5	22.3	32.6	9.0	22.9
22:4n-6	-	0.1	-	1.7	-	0.6	-	0.4	-	0.2	-	0.1	-	0.1
22:5n-3	-	0.1	-	0.3	-	0.7	-	1.0	-	0.4	-	0.3	-	-
22:6n-3	0.8	1.8	1.9	4.2	0.1	2.8	2.5	2.6	1.2	2.4	0.4	0.9	-	1.8

Table S40. The FA composition (%) of polar lipids of three types (L, B and G) zooxanthellae isolated from cnidarians (Okinawa) [115].

Fatty acid	*Millepora intricata*	*Pocillopora damicornis*	*Seriatopora caliendrum*	*Seriatopora hystrix*	*Stylophora pistillata*
	L	B	G	G	B + G
14:0	2.4	2.9	2.7	2.3	2.5
14:1	1.0	0.5	1.0	0.6	0.2
16:0	12.8	16.7	16.6	17.7	18.6
16:1n-7	2.1	2.8	4.3	2.9	2.9
18:0	5.1	7.9	6.9	7.6	6.9
18:1n-9	1.8	1.9	2.5	1.8	2.7
18:1n-7	0.6	1.0	0.8	1.0	0.9

18:2n-6	0.6	1.6	1.7	1.4	1.2
18:3n-6	1.7	2.9	5.2	4.3	1.5
18:3n-3	-	0.5	0.5	0.3	0.4
18:4n-3	26.2	17.5	10.3	10.0	17.2
18:5n-3	8.7	1.3	0.6	0.7	0.8
20:1	0.7	0.7	0.7	1.8	0.9
20:2n-6	0.4	1.1	0.8	0.8	0.8
20:3n-6	0.4	0.6	0.3	0.5	0.4
20:4n-6	0.1	7.5	8.6	11.3	7.5
20:5n-3	0.5	11.2	14.8	10.3	16.1
22:4n-6	4.9	4.6	3.0	3.5	2.1
22:5n-6	10.3	-	-	-	-
22:5n-3	0.5	1.4	0.9	1.1	2.1
22:6n-3	17.8	10.6	16.4	15.9	11.2

Table S41. The FA composition (%) of triacylglycerols of three types (L, B and G) zooxanthellae isolated from cnidarians (Okinawa) [115].

Fatty acid	*Millepora intricata* L	*Pocillopora damicornis* B	*Seriatopora caliendrum* G	*Seriatopora hystrix* G	*Stylophora pistillata* B + G
14:0	2.9	4.6	8.2	8.0	5.1
16:0	26.0	42.3	31.4	32.0	37.2
16:1n-7	1.0	5.8	11.9	12.3	6.2
17:0	0.4	-	0.5	0.2	0.2
18:0	20.9	5.0	4.8	4.4	4.3
18:1n-9	4.2	8.9	8.7	6.1	14.1
18:1n-7	0.4	1.1	1.3	1.3	2.0
18:2n-6	1.2	2.1	3.1	2.0	0.9
18:3n-6	1.8	2.5	0.5	0.3	1.1
18:3n-3	-	0.2	1.0	0.2	0.2
18:4n-3	-	0.2	-	-	0.1
18:5n-3	0.1	-	-	0.3	-
20:1	-	-	-	0.3	-
20:2n-6	0.3	0.1	-	0.6	-
20:3n-6	-	0.1	0.5	-	-
20:4n-6	-	0.4	1.3	0.6	0.5
20:5n-3	-	2.5	4.0	4.5	3.4
22:4n-6	1.0	0.5	0.5	0.4	0.4
22:5n-3	-	0.7	0.5	1.0	1.4
22:6n-3	39.2	21.8	19.3	23.3	21.2

Table S42. The main FA composition (%) of the total lipids in intact colonies, zooxanthellae, and the host tissue of the soft coral *Sinularia* sp. (mean ± SD, *n* = 3) [68].

Lipid class	Intact colonies	Zooxanthellae	Host tissue
14:0*,[a]	2.5 ± 0.3	3.5 ± 0.3	1.4 ± 0.3
14:1	0.2 ± 0.1	1.4 ± 0.3	-
16:0	31.3 ± 4.6	24.7 ± 1.0	28.8 ± 4.1
16:1n-9	0.3 ± 0.1	0.8 ± 0.3	-
16:1n-7	2.1 ± 0.5	1.8 ± 0.0	2.0 ± 0.3
16:2n-7*	4.6 ± 0.6	6.0 ± 0.5	4.2 ± 2.5
16:3n-4**	1.6 ± 0.5	4.5 ± 0.3	1.1 ± 0.3
16:4n-1**	1.4 ± 0.6	4.4 ± 0.5	0.4 ± 0.2
18:0*	9.1 ± 0.7	5.1 ± 0.6	14.3 ± 1.8
18:1n-9	2.2 ± 0.3	2.9 ± 0.1	2.6 ± 0.3
18:1n-7*	0.3 ± 0.1	0.2 ± 0.1	0.7 ± 0.1
18:2n-7**	2.0 ± 0.4	0.8 ± 0.1	4.0 ± 0.7
18:2n-6	0.3 ± 0.1	0.4 ± 0.0	1.4 ± 0.6
18:3n-4*	1.0 ± 0.2	0.3 ± 0.1	1.7 ± 0.2
18:3n-3	0.4 ± 0.1	0.4 ± 0.1	0.4 ± 0.1
18:4n-3**	3.9 ± 0.3	13.3 ± 0.7	1.3 ± 0.2
20:0*	0.6 ± 0.0	0.4 ± 0.1	1.0 ± 0.3
20:3n-6*	0.3 ± 0.1	0.1 ± 0.0	1.0 ± 0.3
20:4n-6**	18.1 ± 5.5	6.7 ± 0.8	14.8 ± 1.7
20:4n-3**	0.9 ± 0.3	0.2 ± 0.1	1.3 ± 0.2
20:5n-3**	2.5 ± 0.3	6.3 ± 0.5	0.6 ± 0.1
22:4n-6	0.4 ± 0.1	0.1 ± 0.0	0.8 ± 0.5
22:6n-3**	2.1 ± 0.6	7.9 ± 0.8	0.9 ± 0.1
24:5n-6**	3.8 ± 1.0	0.8 ± 0.3	5.0 ± 0.9
24:6n-3*	1.2 ± 0.2	0.3 ± 0.2	1.6 ± 0.3

[a] Significant difference between zooxanthellae and the host tissue:* $P < 0.01$, ** $P < 0.01$.

Table S43. Main fatty acid composition (% of total FA) of the total lipids found in the symbiont fractions (SF) and in the host fractions (HF) of cnidarians. Values are means of triplicate samples ± SD [122].

Fatty acid	*Millepora platyphylla*		*Sinularia cf. capitalis*		*Sinularia polydactila*	
	SF	HF	SF	HF	SF	HF
14:0	2.2 ± 0.3	2.0 ± 0.4	6.5 ± 0.1	2.1 ± 0.0	3.3 ± 0.8	1.4 ± 0.3
16:0	18.1 ± 1.1	22.3 ± 2.5	24.5 ± 4.2	38.7 ± 1.7	26.3 ± 3.2	28.8 ± 4.1
16:1n-9	0.5 ± 0.3	0.2 ± 0.0	0.9 ± 0.2	-[a]	0.6 ± 0.1	-
16:1n-7	0.3 ± 0.2	0.2 ± 0.1	1.9 ± 0.1	3.0 ± 0.2	1.9 ± 0.3	2.0 ± 0.3
16:2n-7	-	-	5.7 ± 0.3	3.8 ± 0.7	4.8 ± 0.3	4.1 ± 0.8
16:3n-4	-	-	4.1 ± 0.8	1.1 ± 0.4	4.2 ± 1.6	1.1 ± 0.3
16:4n-1	-	-	3.7 ± 0.8	0.5 ± 0.1	1.5 ± 0.8	0.4 ± 0.2

18:0	7.7 ± 0.9	20.1 ± 0.6	5.7 ± 2.5	14.6 ± 0.3	8.5 ± 1.8	14.3 ± 1.8
18:1n-9	3.4 ± 0.5	3.5 ± 1.2	2.7 ± 0.2	3.1 ± 0.2	2.0 ± 0.3	2.6 ± 0.3
18:1n-7	0.1 ± 0.0	0.1 ± 0.0	0.3 ± 0.1	0.6 ± 0.1	0.3 ± 0.1	0.7 ± 0.1
18:2n-7	-	-	0.9 ± 0.3	2.0 ± 0.1	1.9 ± 0.5	4.0 ± 0.7
18:2n-6	0.3 ± 0.0	0.1 ± 0.0	0.3 ± 0.1	0.9 ± 0.4	0.5 ± 0.2	1.4 ± 0.6
18:3n-6	0.1 ± 0.0	-	0.4 ± 0.1	-	-	0.2 ± 0.1
18:4n-3	17.3 ± 4.5	0.9 ± 0.1	10.6 ± 3.3	1.2 ± 0.0	9.1 ± 2.2	1.3 ± 0.2
20:0	2.5 ± 0.2	4.7 ± 0.3	0.4 ± 0.1	1.3 ± 0.1	0.6 ± 0.2	1.0 ± 0.3
20:1	0.7 ± 0.1	0.2 ± 0.1	0.3 ± 0.0	0.1 ± 0.0	0.2 ± 0.1	-
18:5n-3	4.8 ± 0.5	0.1 ± 0.0	1.5 ± 0.4	0.7 ± 0.3	0.9 ± 0.4	-
20:2n-6	0.8 ± 0.2	-	0.5 ± 0.4	-	0.3 ± 0.1	0.2 ± 0.1
20:3n-6	0.4 ± 0.1	0.2 ± 0.0	0.2 ± 0.0	0.5 ± 0.4	0.6 ± 0.2	1.0 ± 0.3
20:4n-6	0.2 ± 0.1	0.1 ± 0.0	8.1 ± 1.1	12.1 ± 1.6	12.3 ± 3.0	14.8 ± 1.7
20:5n-3	3.4 ± 1.0	0.4 ± 0.1	5.0 ± 1.2	1.2 ± 0.1	4.3 ± 0.8	0.6 ± 0.1
22:4n-6	4.0 ± 0.7	3.5 ± 0.5	0.3 ± 0.1	0.4 ± 0.0	0.3 ± 0.1	0.8 ± 0.5
22:5n-6	5.0 ± 0.2	6.7 ± 1.0	-	-	0.2 ± 0.1	0.3 ± 0.0
22:5n-3	0.6 ± 0.1	0.7 ± 0.1	0.2 ± 0.0	0.4 ± 0.3	0.2 ± 0.1	-
22:6n-3	22.7 ± 1.3	31.7 ± 2.2	7.8 ± 1.2	1.2 ± 0.1	5.3 ± 1.5	0.9 ± 0.1
24:5n-6	-	-	1.5 ± 0.4	3.2 ± 0.6	3.2 ± 1.1	5.0 ± 0.9
24:6n-3	-	-	0.5 ± 0.3	1.0 ± 0.1	0.5 ± 0.2	1.0 ± 0.3

[a] Not detected or less than 0.1%.

Table S43. (*continued*).

Fatty acid	*Acropora intermedia*		*Acropora muricata*		*Montipora digitata*	
	SF	HF	SF	HF	SF	HF
14:0	2.7 ± 0.5	1.9 ± 0.3	2.2 ± 1.7	1.8 ± 0.5	3.0 ± 0.8	3.5 ± 0.1
16:0	18.6 ± 1.7	20.1 ± 3.4	16.0 ± 5.0	17.9 ± 2.4	19.9 ± 2.5	33.7 ± 6.5
16:1n-9	1.5 ± 0.6	1.2 ± 0.4	0.8 ± 0.3	1.3 ± 0.9	4.1 ± 0.7	1.2 ± 0.2
16:1n-7	1.9 ± 0.5	1.1 ± 0.2	2.7 ± 0.3	1.9 ± 1.1	2.7 ± 2.2	2.9 ± 0.7
16:2n-7	-	0.2 ± 0.1	0.9 ± 0.4	0.1 ± 0.0	0.8 ± 0.8	0.2 ± 0.1
16:3n-4	-	-	-	-	-	-
16:4n-1	-	-	-	-	-	-
18:0	5.4 ± 1.2	13.6 ± 2.6	3.5 ± 1.5	18.2 ± 2.6	3.0 ± 0.9	7.7 ± 1.6
18:1n-9	2.9 ± 0.4	3.0 ± 0.4	2.7 ± 0.4	3.3 ± 0.6	2.2 ± 0.9	2.9 ± 2.0
18:1n-7	0.6 ± 0.6	0.3 ± 0.0	0.3 ± 0.3	0.4 ± 0.0	0.1 ± 0.0	0.3 ± 0.1
18:2n-7	0.1 ± 0.0	-	0.1 ± 0.0	-	-	0.1 ± 0.0
18:2n-6	1.0 ± 0.4	0.6 ± 0.1	1.1 ± 0.1	0.9 ± 0.2	1.0 ± 0.3	1.3 ± 0.1
18:3n-6	8.2 ± 0.5	2.7 ± 0.8	11.4 ± 0.1	2.7 ± 1.0	17.2 ± 2.8	3.6 ± 1.9
18:4n-3	13.0 ± 0.9	0.9 ± 0.3	14.1 ± 1.0	1.3 ± 0.6	15.0 ± 5.4	1.3 ± 0.6
20:0	0.3 ± 0.0	2.2 ± 0.4	0.4 ± 0.3	0.9 ± 0.0	0.2 ± 0.1	0.4 ± 0.1
20:1	1.3 ± 0.2	2.8 ± 0.4	0.9 ± 0.2	3.6 ± 0.3	1.0 ± 0.1	0.2 ± 0.1
18:5n-3	2.8 ± 0.4	0.2 ± 0.1	2.6 ± 0.7	0.2 ± 0.1	5.1 ± 1.4	0.1 ± 0.0

20:2n-6	2.0 ± 1.4	0.4 ± 0.1	2.6 ± 1.7	0.4 ± 0.1	0.5 ± 0.7	0.5 ± 0.1
20:3n-6	0.4 ± 0.1	1.0 ± 0.1	0.4 ± 0.1	0.8 ± 0.1	0.5 ± 0.2	1.6 ± 0.2
20:4n-6	5.3 ± 0.8	17.2 ± 3.1	2.9 ± 1.5	13.7 ± 1.2	3.4 ± 1.6	9.9 ± 2.1
20:5n-3	14.7 ± 1.6	8.7 ± 1.8	15.8 ± 3.8	10.2 ± 0.7	6.7 ± 2.5	1.9 ± 0.5
22:4n-6	2.3 ± 0.4	8.1 ± 1.6	1.2 ± 0.4	8.1 ± 1.3	4.8 ± 2.4	22.5 ± 7.4
22:5n-6	-	-	-	0.1 ± 0.0	-	-
22:5n-3	2.1 ± 0.3	4.3 ± 0.2	1.2 ± 0.5	4.0 ± 0.6	1.3 ± 0.6	2.1 ± 0.9
22:6n-3	8.4 ± 1.1	5.0 ± 0.3	11.4 ± 3.4	3.6 ± 0.6	6.9 ± 1.6	1.0 ± 0.6
24:5n-6	-	-	-	-	-	-
24:6n-3	-	-	-	-	-	-

Table S43. (*continued*).

Fatty acid	*Montipora foliosa*		*Pavona decussata*		*Pocillopora damicornis*		*Porites cylindrica*	
	SF	HF	SF	HF	SF	HF	SF	HF
14:0	3.0 ± 0.3	2.9 ± 0.1	2.1 ± 0.4	5.4 ± 0.7	2.9 ± 0.5	3.3 ± 0.3	3.5 ± 0.3	3.1 ± 1.3
16:0	18.5 ± 1.3	29.4 ± 0.9	15.9 ± 1.4	27.4 ± 3.8	21.7 ± 2.2	31.9 ± 0.9	16.3 ± 1.6	30.1 ± 5.6
16:1n-9	1.9 ± 0.7	1.5 ± 0.4	0.2 ± 0.1	0.3 ± 0.0	0.5 ± 0.1	-	0.5 ± 0.1	1.1 ± 0.4
16:1n-7	2.0 ± 0.1	1.8 ± 0.1	1.7 ± 0.2	4.5 ± 0.6	4.5 ± 0.3	3.5 ± 0.3	1.1 ± 0.2	2.6 ± 1.3
16:2n-7	0.7 ± 0.4	0.1 ± 0.0	-	0.1 ± 0.0	0.9 ± 0.3	0.2 ± 0.0	1.6 ± 0.4	0.2 ± 0.1
16:3n-4	-	-	-	-	-	-	-	-
16:4n-1	-	-	-	-	-	-	-	-
18:0	2.5 ± 0.6	8.7 ± 0.4	2.8 ± 0.7	19.0 ± 2.9	5.2 ± 0.2	8.2 ± 0.5	4.7 ± 0.8	10.1 ± 2.5
18:1n-9	4.0 ± 0.8	7.3 ± 0.9	1.4 ± 0.3	7.9 ± 1.5	2.6 ± 0.5	6.1 ± 0.2	3.3 ± 0.5	5.1 ± 2.3
18:1n-7	0.5 ± 0.4	0.3 ± 0.1	0.2 ± 0.1	4.0 ± 1.1	0.3 ± 0.1	0.5 ± 0.0	0.2 ± 0.0	0.4 ± 0.2
18:2n-7	0.1 ± 0.1	-	0.1 ± 0.0	0.3 ± 0.1	-	0.1 ± 0.0	0.3 ± 0.0	0.1 ± 0.0
18:2n-6	1.0 ± 0.2	2.4 ± 1.2	0.8 ± 0.2	0.9 ± 0.2	1.0 ± 0.1	1.3 ± 0.1	0.8 ± 0.2	1.1 ± 0.7
18:3n-6	23.4 ± 1.4	2.4 ± 0.4	12.4 ± 1.2	0.3 ± 0.0	4.8 ± 0.2	4.4 ± 0.3	8.2 ± 0.7	2.8 ± 1.1
18:4n-3	10.3 ± 0.8	0.6 ± 0.2	29.5 ± 2.7	1.3 ± 0.4	10.3 ± 0.9	0.7 ± 0.1	13.1 ± 2.1	0.9 ± 0.3
20:0	0.2 ± 0.1	0.4 ± 0.0	0.3 ± 0.1	-	0.8 ± 0.0	1.5 ± 0.1	0.2 ± 0.1	1.0 ± 0.7
20:1	0.9 ± 0.1	0.4 ± 0.1	0.2 ± 0.1	0.6 ± 0.3	1.2 ± 0.3	1.0 ± 0.1	0.6 ± 0.2	1.7 ± 0.4
18:5n-3	5.6 ± 0.2	0.4 ± 0.2	2.3 ± 0.4	0.8 ± 0.2	2.3 ± 0.3	0.1 ± 0.0	3.9 ± 0.5	0.2 ± 0.1
20:2n-6	0.5 ± 0.6	0.6 ± 0.1	0.7 ± 0.2	-	0.5 ± 0.3	0.6 ± 0.0	1.2 ± 0.3	0.5 ± 0.1
20:3n-6	0.2 ± 0.0	1.1 ± 0.1	0.2 ± 0.0	2.5 ± 0.7	2.1 ± 0.2	5.6 ± 0.4	-	1.8 ± 0.9
20:4n-6	2.3 ± 0.5	11.2 ± 1.2	4.2 ± 0.9	10.3 ± 1.9	6.2 ± 0.4	8.8 ± 0.7	8.0 ± 1.3	10.2 ± 1.7
20:5n-3	5.1 ± 0.6	1.9 ± 0.2	7.7 ± 0.7	1.7 ± 0.3	14.2 ± 1.2	3.1 ± 0.1	7.7 ± 1.1	4.4 ± 0.4
22:4n-6	3.6 ± 0.3	20.2 ± 1.9	1.3 ± 0.4	6.4 ± 2.3	3.0 ± 0.2	5.1 ± 0.9	3.4 ± 0.4	10.7 ± 2.7
22:5n-6	-	-	-	-	-	-	-	0.1 ± 0.0
22:5n-3	0.6 ± 0.0	2.0 ± 0.2	1.4 ± 0.3	0.5 ± 0.1	0.8 ± 0.2	1.2 ± 0.0	1.9 ± 0.2	2.4 ± 1.5
22:6n-3	7.7 ± 1.7	1.2 ± 0.0	9.2 ± 0.9	2.8 ± 0.8	9.8 ± 0.5	9.6 ± 0.1	9.2 ± 1.5	3.6 ± 1.4
24:5n-6	-	-	-	-	-	-	-	-
24:6n-3	-	-	-	-	-	-	-	-

Table S44. Hard coral species from Vietnam and Seychelles used for statistical analysis [103].

N	Family	Species	Region	Sampling site
1	Acroporidae	*Acropora nasuta**	Vietnam	The Tyam Island
2		*Acropora nasuta**	Vietnam	The Thotyu Island
3		*Acropora millepora**	Vietnam	The Tyam Island
4		*Acropora millepora**	Vietnam	The Thotyu Island
5		*Acropora florida**	Vietnam	The Thotyu Island
6		*Acropora cerealis*	Vietnam	The Mun Island
7		*Acropora Formosa*	Vietnam	The Mun Island
8		*Acropora gemmifera*	Vietnam	The Mun Island
9		*Acropora palifera*	Vietnam	The Mun Island
10		*Acropora* sp.	Vietnam	The Nha Trang Bay
11		*Acropora nobilis*	Vietnam	The Nha Trang Bay
12	Pocilloporidae	*Seriatopora caliendrum**	Сейшелы	The Aldabra Island
13		*Seriatopora hystrix*	Vietnam	The Mun Island
14		*Stylophora pistillata**	Seychelles	The Coetivy Island
15		*Stylophora pistillata**	Vietnam	The Tyam Island
16		*Stylophora pistillata**	Vietnam	The Thotyu Island
17		*Stylophora pistilata*	Vietnam	The Mun Island
18		*Pocillopora damicornis**	Vietnam	The Thotyu Island
19		*Pocillopora damicornis**	Vietnam	The Thotyu Island
20		*Pocillopora damicornis*	Vietnam	The Mun Island
21		*Pocillopora verrucosa**	Vietnam	The Thotyu Island
22	Pectiniidae	*Echinophyllia orpheensis*	Vietnam	The Nha Trang Bay
23	Fungiidae	*Sandalolitha robusta*	Vietnam	The Nha Trang Bay
24	Poritidae	*Goniopora* sp. I*	Vietnam	The Tyam Island
25		*Goniopora* sp. II*	Vietnam	The Tyam Island
26		*Porites cylindrica*	Vietnam	The Mun Island
27		*Porites nigrescens*	Vietnam	The Mun Island
28		*Porites lobata*	Vietnam	The Nha Trang Bay
29	Faviidae	*Favia* sp. I	Vietnam	The Nha Trang Bay
30		*Favia* sp. II	Vietnam	The Nha Trang Bay
31	Dendrophylliidae	*Tubastrea coccinea**	Сейшелы	The Aldabra Island
32		*Tubastrea micrantha**	Seychelles	The Aldabra Island
33	Milleporidae	*Millepora sp.**	Vietnam	The Thotyu Island

34		*Millepora platyphylla**	Seychelles	The Aldabra Island
35		*Millepora dichotoma**	Seychelles	The Aldabra Island

* Data on FA composition from Latyshev et al., 1991.

Table S45. The main FA composition (% of total) of heterotrophic bacteria, associated with colonies of 11 health hard coral species (of the genera *Montipora*, *Porites*, *Acropora*, and *Hydnophora*) collected in Nha Trang Bay (Vietnam) [213].

Fatty acid	*Pseudomonas*			*Halomonas*		*Bacillus*	*Planococcus*	*Micrococcus*	*Vibrio*
	344	368	374	350	369	356	362	370	366
10:0	3.2	–	0.7	2.8	1.1	–	–	–	–
12:0	4.6	3.4	1.5	3.6	1.1	–	–	–	3.9
i-13:0	–	–	–	–	–	–	–	0.6	0.5
ai-13:0	–	–	–	–	–	–	–	2.5	–
i-14:0	–	–	–	–	–	0.9	2.4	4.3	–
14:0	0.1	0.1	0.3	0.2	3.2	0.5	0.5	5.1	0.7
i-15:0	–	–	–	–	–	6.3	12.1	8.5	–
ai-15:0	–	–	–	–	–	60.0	44.9	75.3	0.6
15:0	0.1	–	0.1	–	–	0.6	2.4	2.3	3.2
15:1n-8	–	–	–	–	–	–	–	0.8	1.3
i-16:0	–	–	–	–	–	5.0	1.9	1.5	0.9
16:0	22.2	23.1	18.4	20.7	21.8	12.8	0.7	0.4	14.2
i-16:1	–	–	–	–	–	–	6.5	0.8	–
16:1n-7	44.1	42.3	9.7	33.2	17.5	–	–	0.9	42.0
16:1n-9	–	–	–	–	–	–	3.1	–	–
i-17:0	–	–	–	–	–	–	3.2	0.9	–
ai-17:0	–	–	–	–	–	10.6	11.5	–	–
17:0	0.1	–	0.1	–	–	–	0.8	–	1.6
i-17:1	–	–	–	–	–	–	2.8	–	–
ai-17:1	–	–	–	–	–	–	4.9	–	–
17:1n-8	0.2	–	–	–	–	–	–	0.8	2.5
cy-17:0	4.2	3.3	6.2	–	–	–	–	0.5	
i-18:0	–	–	–	–	–	–	0.5	–	–
18:0	–	–	0.1	0.4	–	–	0.3	–	–
18:1n-9	–	–	–	–	–	–	–	–	–
18:1n-7	20.1	24.8	36.4	38.2	57.8	–	1.1		23.2
cy-19:0	–		25.8	–	–	–	–	–	–
